# Stoichiometrically Defined Antibody–DNA Conjugates for Quantitative Super-Resolution Imaging

**DOI:** 10.1021/acs.nanolett.6c02173

**Published:** 2026-05-20

**Authors:** Luciana P. Martinez, Clíona McMahon, Cecilia Zaza, Olivia P. L. Dalby, Callum J. Stack, James R. Baker, Vijay Chudasama, Sabrina Simoncelli

**Affiliations:** † London Centre for Nanotechnology, 4919 University College London, 19 Gordon Street, WC1H 0AH London, United Kingdom; ‡ Department of Chemistry, 4919 University College London, 20 Gordon Street, WC1H 0AJ London, United Kingdom

**Keywords:** pyridazinedione chemistry, site-selective labeling, stoichiometric antibody−DNA conjugates, single-molecule localization microscopy, quantitative DNA-PAINT

## Abstract

DNA-PAINT enables super-resolution imaging with molecular-scale precision and, through qPAINT analysis, quantitative determination of protein copy numbers. However, qPAINT accuracy critically depends on the stoichiometric definition of the DNA–antibody conjugate. Conventional amine-reactive labeling generates heterogeneous antibody populations carrying variable numbers of DNA docking strands, compromising quantitative reliability. Here, we introduce site-selective disulfide rebridging using pyridazinedione chemistry to generate stoichiometrically defined antibody–DNA conjugates with either one (OAR1) or four (OAR4) DNA-PAINT docking sequences per antibody. Using nuclear pore complexes as a benchmark, we demonstrate that both OAR1 and OAR4 probes yield narrow qPAINT index distributions that exhibit the expected proportional dependence on protein copy number, enabling reliable discrimination between singly and doubly labeled Nup96 dimers. In contrast, conventional lysine-based conjugation produces broadened distributions and systematic overestimation due to uncontrolled DNA loading. These results establish selective antibody–DNA conjugation as a prerequisite for accurate quantitative DNA-PAINT in complex cellular environments.

## Graphical abstract

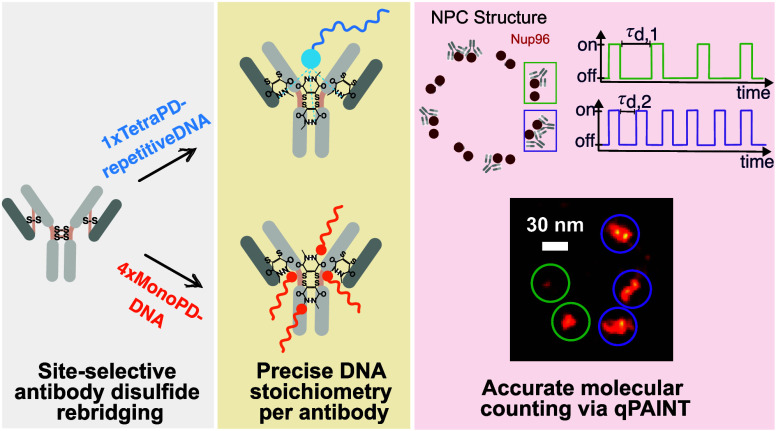

DNA-PAINT (DNA Points Accumulation for Imaging in Nanoscale Topography) has established itself as a powerful super-resolution microscopy technique that enables imaging with molecular-level precision.^1^ In DNA-PAINT, short fluorescently labeled “imager” strands transiently hybridize to complementary “docking” strands that are covalently attached to the target of interest. The stochastic nature of these binding events produces a sequence of fluorescence bursts whose frequency directly reflects the number of docking sites.^2,3^ Through qPAINT (quantitative PAINT) analysis, the kinetics of these binding interactions can be used to infer the number of target molecules transforming super-resolution microscopy into a truly quantitative molecular tool.^4^ Recent technological developments in DNA-PAINT have primarily focused on enabling multiplexed imaging^5^ and accelerated acquisition strategies.^6,7^ By contrast, the stoichiometric definition of labeling probes has received comparatively less attention, despite being critical for quantitative approaches such as qPAINT, which rely on the precise interpretation of binding kinetics to achieve accurate molecular counting.

The quantitative accuracy of DNA-PAINT depends critically on the stoichiometric definition of the DNA–antibody conjugate. However, for the vast majority of endogenous targets, DNA-PAINT relies on full-length IgG antibodies, for which broadly applicable strategies to achieve defined antibody-DNA stoichiometry remain limited. The most widely used approaches to attach DNA to antibodies rely on nonselective amine-reactive chemistries, such as maleimide–PEG_2_–NHS or DBCO–sulfo–NHS linkers,^8^ which target lysine residues distributed across the antibody surface. This results in heterogeneous conjugates that can carry anywhere from zero to several DNA strands per antibody. Such uncontrolled labeling reduces the proportion of functional antibodies and introduces uncertainty into the relationship between observed qPAINT kinetics and actual protein numbers. When each antibody carries a variable number of DNA docking strands, the proportional relationship between inverse dark time and target copy number is no longer preserved, and protein number estimates lose precision. Although many studies have demonstrated molecular counting via qPAINT,^9–16^ a robust and chemically defined strategy to generate antibody–DNA conjugates with precisely controlled stoichiometry have been lacking.

To address this limitation, in this work, we develop and validate a site-selective antibody–DNA conjugation strategy based on disulfide rebridging using dibromopyridazinedione chemistry, which yields stoichiometrically defined probes with precisely controlled oligonucleotide-to-antibody ratios, and we demonstrate their impact on quantitative qPAINT imaging using the nuclear pore complex as a benchmark system.

To generate antibody-DNA conjugates, with defined stoichiometry, we employed site-selective disulfide rebridging using dibromopyridazinedione (PD) linkers to modify the four native interchain disulfide bonds of IgG1 antibodies in a controlled manner. This strategy enabled the preparation of monodisperse antibody–DNA conjugates carrying either exactly four nonrepetitive DNA docking strands per antibody (i.e., an oligonucleotide-to-antibody ratio of 4, OAR4) or exactly one 4x repetitive DNA-PAINT docking strand^17^ per antibody (OAR1) in a facile and modular manner. For the synthesis of the OAR4 conjugate, we utilized a mono-PD containing a bicyclononyne (BCN) click-handle (BCN PD), allowing us to leverage our previously optimized site-specific disulfide rebridging protocol (Figure 1a).^18,19^ Based on our previous observations that the conjugation of “bulky” modules to PD linkers is more favorable when the click reaction is performed before antibody modification, we first coupled a tetrazine-functionalized oligonucleotide (5′-ACCACCA-3′) to BCN PD to afford a “pre-clicked” construct **1**. Subsequent reaction with a reduced anti-GFP antibody yielded the desired OAR4 conjugate **2** (Figure S1–S2).

**Figure 1:**
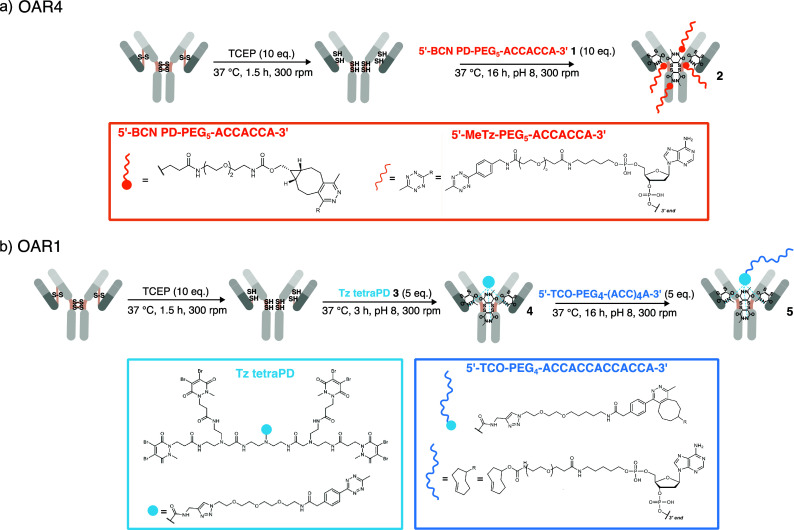
Site-selective disulfide rebridging enables stoichiometrically defined antibody–DNA conjugates. (a) Schematic of the OAR4 conjugation strategy. The four native interchain disulfide bonds of a reduced IgG are rebridged using a bicyclononyne-functionalized monopyridazinedione (BCN PD). Prior to antibody modification, the BCN–PD is reacted with a tetrazine-functionalized DNA-PAINT docking strand (MeTz-DNA), yielding a preclicked PD–DNA construct that installs four nonrepetitive docking strands per antibody upon rebridging. (b) Schematic of the OAR1 conjugation strategy. A tetrazine-functionalized tetra-pyridazinedione (Tz tetra-PD) scaffold bearing a single click handle simultaneously rebridges all four reduced interchain disulfides of the antibody, generating a single, site-defined attachment point. Subsequent reaction with a TCO-functionalized repetitive DNA-PAINT docking strand (TCO–DNA) yields an antibody carrying one 4× repetitive docking sequence (OAR1).

While PD-based reagents enabling antibody conjugates with payload-to-antibody ratios of two or four had been previously reported,^18–20^ no equivalent strategy existed to generate a strictly monovalent antibody-DNA conjugate. Inspired by the work of Dannheim et al.,^21^ we designed a tetrazine-functionalized tetra-PD scaffold **3**, in which four PD units are linked together and share a single click-handle (Figure 1b) (see Supporting Information for full details). This reagent rebridges all four reduced disulfide bonds of the antibody simultaneously, creating a single attachment site for one DNA docking strand. As only one oligonucleotide was to be attached per antibody in this construct, conjugation could be performed either before or after PD disulfide rebridging of the antibody. To demonstrate postconjugation click compatibility, the tetra-PD **3** was first used to rebridge the reduced anti-GFP antibody, forming conjugate **4** (Figure S3), which was subsequently clicked with a *trans*-cyclooctene (TCO)-bearing oligonucleotide (5′-ACCACCACCACCACCA-3′) via inverse electron demand strain-promoted cycloaddition (iEDDA-SPAAC) to afford OAR1 conjugate **5** (Figure S4). Both conjugation strategies yielded reproducible antibody-DNA conjugates, as verified by UV–vis absorbance and mass spectrometry (Figures S1–S5), which confirmed the incorporation of the expected number of oligonucleotide strands per antibody molecule. To further assess the distribution of antibody assembly states, we performed SDS–PAGE analysis under nonreducing conditions (Figure S6). These experiments confirmed the mass spectrometry observations that OAR1 is predominantly present as a full-length antibody conjugate, whereas OAR4 consisted of a mixture of full-length and half antibody conjugates. This distribution is consistent with native antibody disulfide rebridging strategies and does not appear to compromise antigen binding.^22^ In fact, for the PD-based reagents used in this manuscript, this has been demonstrated in previous studies with a model PD-based compound^23^ as well as in various PD-based antibody-conjugates.^24–27^ In contrast, conjugation of anti-GFP antibody with maleimide–PEG_2_–NHS (followed by reaction with 5′-thiol-AAACCACCACCACCACCA-3′), one of the standard lysine/amine-reactive methods used throughout the DNA-PAINT community, resulted in heterogeneous products with variable DNA-to-antibody ratios (Figure S7; anti-GFP–lysine–oligo conjugate **6**, average OAR ∼ 3.7 as determined via UV–vis spectroscopy, Figure S5), consistent with random modification of accessible lysine residues. To benchmark the performance of the site-selective antibody–DNA conjugates in a cellular context, we imaged nuclear pore complexes (NPCs) in U2OS cells expressing Nup96–GFP. The NPC is a large, multiprotein assembly spanning the nuclear envelope that mediates the selective exchange of macromolecules between the cytoplasm and nucleus. Owing to its highly stereotypic and symmetric architecture, the NPC serves as an established reference structure for assessing spatial resolution and molecular counting accuracy in super-resolution microscopy.^28^

First, we compared three labeling strategies: (i) the conventional anti-GFP nanobody conjugated to a single, repetitive DNA-PAINT docking sequence (the standard commercially available DNA-PAINT probe used for GFP-labeled proteins),^29^ (ii) the OAR4 conjugate carrying four nonrepetitive short docking strands per antibody, and (iii) the OAR1 antibody–DNA conjugate carrying one repetitive 4x docking strand per antibody. Figure 2a shows representative DNA-PAINT images of the nuclear envelope and zoomed-in regions highlighting individual NPCs obtained with each labeling strategy. All approaches yielded the expected ring-like assemblies distributed across the nuclear envelope. The inset in Figure 2a (left) schematically depicts the 8-fold rotational symmetry of the NPC, where Nup96 proteins form stable homodimeric pairs arranged on the cytoplasmic and nucleoplasmic rings. The localization precision, estimated by the Cramér–Rao Lower Bound (CRLB) and the nearest-neighbor analysis (NeNA),^30^ was less than 3 and 4 nm, respectively, for all conditions (see Table S1), indicating comparable single-molecule localization accuracy across labeling strategies. Furthermore, the number of localizations per NPCs per minute was comparable for both OAR4 and OAR1 labeling conditions (11 ± 2 and 14 ± 3, respectively, for 0.3 nM imager concentration), confirming that increasing the number of attached DNA strands per antibody—from one 4x repetitive sequence to four 1x sequence—does not measurably alter binding rates within the imaging regime as expected.^17^ Localization per minute for all conditions can be found in Table S1.

**Figure 2:**
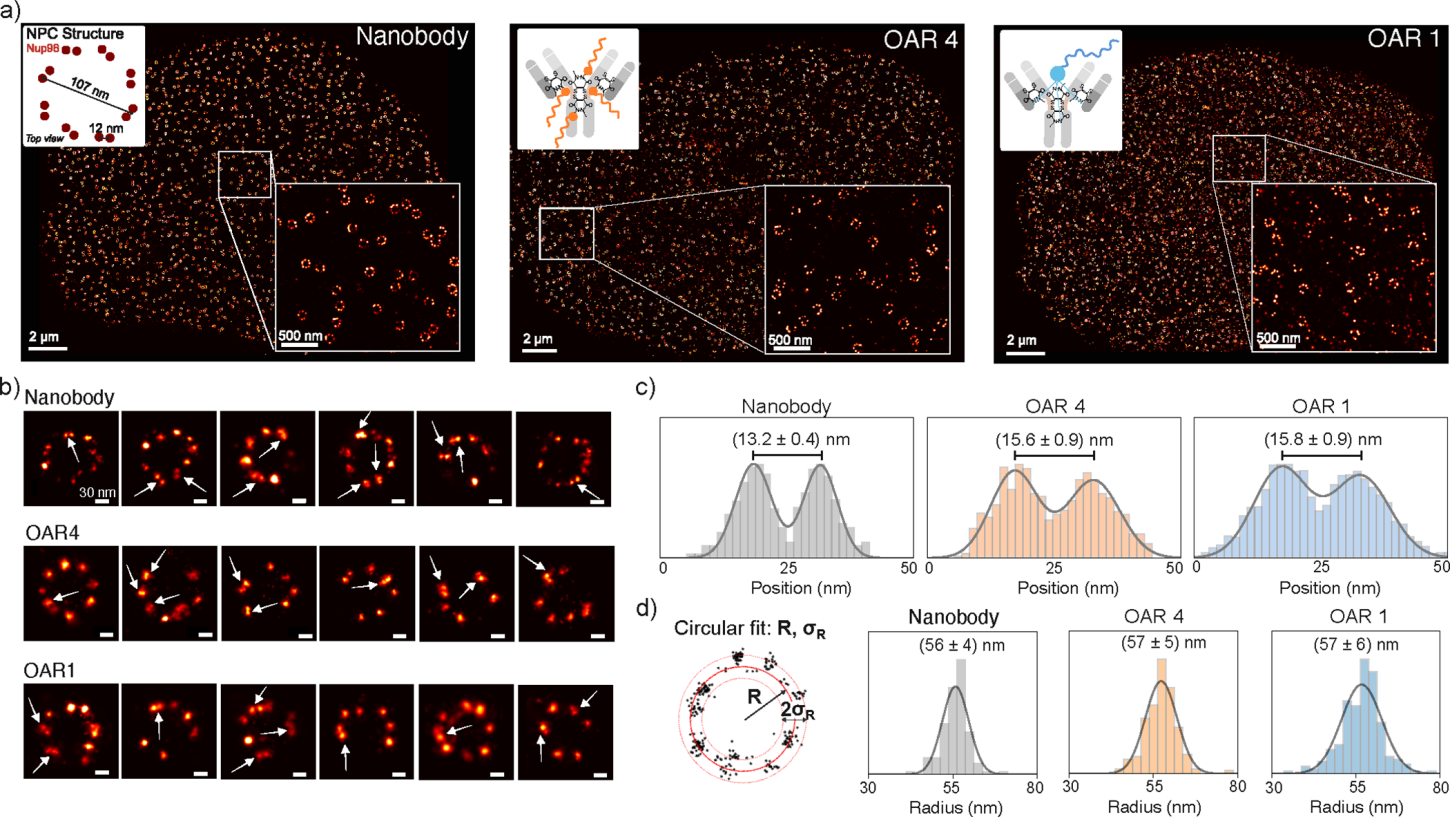
DNA-PAINT imaging of nuclear pore complexes using stoichiometrically defined antibody–DNA conjugates. (a) Representative DNA-PAINT images of the nuclear envelope in U2OS cells expressing Nup96–GFP, labeled using three strategies: a conventional anti-GFP nanobody carrying a single repetitive DNA-PAINT docking strand (left), an OAR4 full-length anti-GFP antibody carrying four nonrepetitive docking strands (middle), and an OAR1 full-length anti-GFP antibody carrying one 4× repetitive docking sequence (right). Zoomed-in regions highlight individual nuclear pore complexes (NPCs) exhibiting the characteristic ring-like architecture. A schematic illustrating the 8-fold rotational symmetry and dimeric organization of Nup96 within the NPC is shown as an inset in the left panel. (b) Magnified views of individual NPCs from each labeling condition, revealing paired localization features corresponding to Nup96 homodimers (arrows). All labeling strategies resolve the dimeric substructure of the NPC. (c) Cross-sectional intensity profiles averaged over aligned Nup96 dimers. The nanobody-based probe yields an interdimer separation of (13.2 ± 0.4) nm (*n* = 20), whereas full antibody labeling results in dimer separations of (15.6 ± 0.9) nm (OAR4, *n* = 10) and (15.8 ± 0.9) nm (OAR1, *n* = 20). (d) Distributions of NPC ring radii measured for nanobody, OAR4, and OAR1 labeling.

Closer inspection of individual NPCs (Figure 2b) revealed distinct pairs of closely spaced Nup96 proteins corresponding to the dimeric organization within each 8-fold ring. These paired features were clearly resolved for all three labeling methods, as indicated by arrows in Figure 2b, confirming that the OAR1 and OAR4 conjugates retain full target specificity and spatial resolving capability comparable to the nanobody standard. Cross-sectional analysis of aligned Nup96 pairs (Figure 2c) revealed interdimer distances that depended on the labeling strategy. For the nanobody-based probe, the measured separation was (13.2 ± 0.4) nm, consistent with previously reported values obtained by cryo-EM and single-molecule localization microscopy.^31^ In contrast, labeling with full-length anti-GFP antibodies resulted in larger apparent interdimer distances of (15.6 ± 0.9) nm and (15.8 ± 0.9) nm for the OAR4 and OAR1 conjugates, respectively. This systematic increase is readily explained by the larger physical size and extended binding geometry of full antibodies compared to nanobodies, which display the DNA docking strands further from the GFP epitope. Importantly, the close agreement between OAR1 and OAR4 measurements indicates that the disulfide rebridging conjugation strategy does not introduce additional structural bias beyond the intrinsic antibody size. These results demonstrate that, while probe geometry influences absolute distance measurements, site-specifically conjugated full antibodies preserve the underlying NPC architecture and enable reliable, internally consistent spatial measurements in DNA-PAINT imaging.

To evaluate potential differences in the apparent NPC geometry arising from probe size or labeling configuration, we further measured the radii of individual NPC rings (Figure 2d). The distributions obtained for nanobody, OAR4, and OAR1 labeling were nearly identical, with mean radii of (56 ± 4) nm, (57 ± 5) nm, and (57 ± 6) nm, respectively, in agreement with previously published values for Nup96–GFP structures.^28^ Figure S8a shows five representative NPCs for each labeling condition, illustrating the dispersion of localizations around the fitted radius at the single-NPC level. This highlights the range of radial deviations present within individual NPCs. In addition to these examples, we quantified the radial spread (σ_R_) of the circular fits across all NPCs (Figure S8b) to assess potential differences in localization dispersion. Antibody-based labeling exhibited a modestly broader radial distribution (σ_R_ ∼ 16–17 nm) compared to the nanobody probe (σ_R_ ∼ 11 nm), consistent with the slight increase also observed for the interdimer distances. Importantly, this broader spread did not result in a measurable expansion of the overall NPC radius. Accordingly, no significant expansion of the apparent NPC size was observed for the antibody-based conjugates relative to the nanobody, indicating that both the OAR1 and OAR4 chemistries preserve the native architecture of the complex. Together, these results demonstrate that stoichiometrically defined OAR1 and OAR4 conjugates perform equivalently to conventional nanobody-based labeling in terms of structural fidelity.

Next, to evaluate labeling performance for quantitative measurements, we performed qPAINT analysis on individual pairs of Nup96 dimers within single NPCs imaged under conditions optimized for kinetic measurements (see Table S1). The qPAINT index is defined as the inverse of the mean dark time between consecutive imager binding events and is therefore proportional to the number of accessible binding sites within a localization cluster. Accurate molecular counting by qPAINT critically depends on homogeneous DNA labeling stoichiometry per antibody. For site-selective conjugation (OAR1 and OAR4), each antibody carries a defined number of docking strands. Under these conditions, the dark time serves as a direct proxy for the number of antibody-labeled proteins within a cluster of single-molecule localizations. A monomer displays a characteristic dark time (τ_d1_), whereas two labeled proteins (i.e., a dimer) exhibits half of that value (τ_d2_), reflecting the doubled probability of imager binding (Figure 3a, central panel). In contrast, nonselective conjugation introduces stochastic variability in the number of binding sites per antibody. Because the labeling stoichiometry is variable, the measured resulting dark times reflect the total number of DNA binding sites rather than the true number of proteins. In this scenario, the kinetic signature scales with binding site abundance, complicating discrimination between monomeric and dimeric protein states (Figure 3a, right panel).

**Figure 3:**
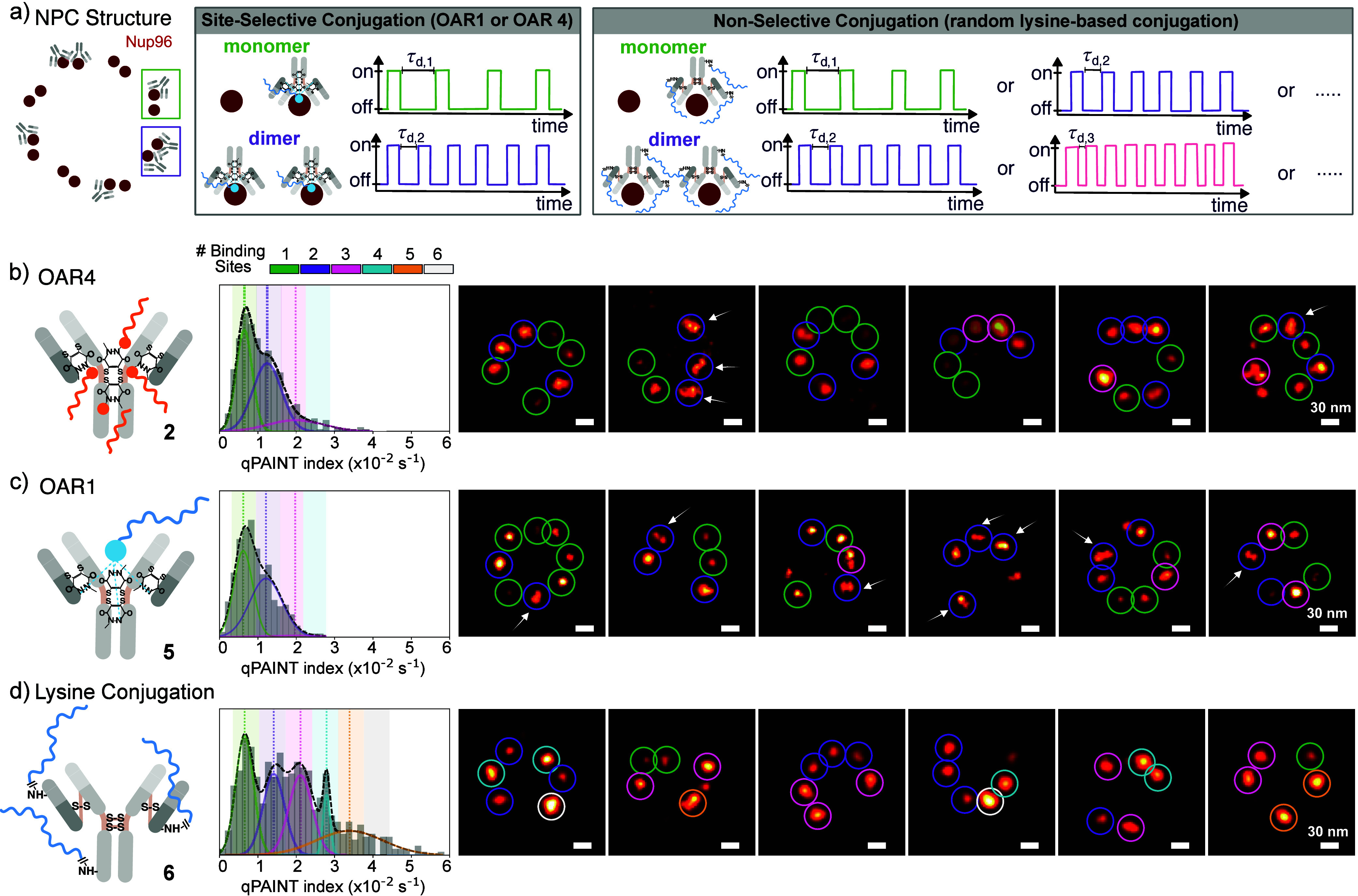
qPAINT analysis reveals improved quantitative precision with stoichiometrically defined antibody–DNA conjugates. (a) Schematic illustrating qPAINT behavior of Nup96–GFP within the 8-fold symmetric nuclear pore complex (NPC). Each NPC contains eight symmetry-related Nup96 positions, each occupied by a stable homodimer. Due to incomplete labeling efficiency, individual sites may be singly labeled (monomeric), doubly labeled (dimeric), or undetected. qPAINT indices were extracted independently from each symmetry position using cluster analysis. (b–d) qPAINT index histograms obtained from NPCs in U2OS cells expressing Nup96–GFP, labeled with three different antibody–DNA conjugation strategies: OAR4 anti-GFP antibodies carrying four nonrepetitive docking strands (top), OAR1 anti-GFP antibodies carrying one 4× repetitive docking sequence (middle), and random lysine-based conjugation of anti-GFP antibodies carrying a variable number of 4× repetitive docking sequences (bottom). OAR4 and OAR1 labeling yield narrow distributions with two well-defined populations corresponding to singly and doubly labeled Nup96 sites (qPAINT indices distributions centered at μ_1_ = 0.64 × 10^–2^ s^–1^ and μ_2_ = 1.24 × 10^–2^ s^–1^; and μ_1_ = 0.62 × 10^–2^ s^–1^ and μ_2_ = 1.21 × 10^–2^ s^–1^, respectively), whereas random lysine conjugation produces a broadened distribution extending to higher apparent qPAINT indices (μ_1_ = 0.65 × 10^–2^ s^–1^, μ_2_ = 1.41 × 10^–2^ s^–1^, μ_3_ = 2.11 × 10^–2^ s^–1^, μ_4_ = 2.79 × 10^–2^ s^–1^, μ_5_ = 3.39 × 10^–2^ s^–1^). Representative NPCs illustrating qPAINT-based classification of individual Nup96 dimer sites. Crosses mark analyzed positions; green indicates monomeric labeling, purple indicates dimeric labeling, and black indicates higher apparent labeling states. For OAR4- and OAR1-labeled NPCs (top and middle), qPAINT classification is consistent with visual inspection of localization density. In contrast, random lysine-based labeling (bottom) yields occasional sites classified as higher-order states (trimers or tetramers) despite identical underlying protein stoichiometry, reflecting conjugation-induced heterogeneity.

To apply this kinetic framework to the biological system, the analysis was performed in two sequential steps: (i) spatial clustering and (ii) temporal trace analysis (see Supporting Information for a detailed description). Spatial clustering was performed independently around each of the eight anticipated Nup96 pair positions within individual NPCs, without imposing any symmetry constraints. qPAINT indices were then extracted from the resulting clusters. Due to incomplete antibody labeling, the analyzed population consists of a mixture of single-antibody-labeled dimers (manifesting as monomeric-like events) and double-antibody-labeled dimers (Figure 3a).

As a result, the qPAINT histograms reflect a mixture of these two antibody-labeling states, allowing us to assess the precision and stoichiometric uniformity of each DNA-conjugation strategy at the single-pair level. For the OAR4 and OAR1 conjugates, the extracted qPAINT histograms revealed two well-defined populations corresponding to Nup96 pairs labeled with either one or two DNA-conjugated antibodies (Figure 3b-c). The resulting distributions were narrow and nearly identical in width for both conjugation strategies, and their means were separated by a factor of approximately two, consistent with the expected difference between singly and doubly labeled Nup96 dimers (see Table S2). In fact, the close agreement between the qPAINT index distributions obtained for OAR1 and OAR4 conjugates suggests that the half antibody conjugate species detected via SDS-PAGE for OAR4 remains functionally competent and presents an effective number of accessible DNA docking strands comparable to the full antibody under imaging conditions. Specifically, the distributions of the extracted qPAINT indices were centered at μ_1_ = 0.64 × 10^–2^ s^–1^ and μ_2_ = 1.24 × 10^–2^ s^–1^ for OAR4 and at μ_1_ = 0.62 × 10^–2^ s^–1^ and μ_2_ = 1.21 × 10^–2^ s^–1^ for OAR1, respectively, confirming the expected proportionality between qPAINT index and protein copy number. A calibration value, $\overset{®}{\mu_{1}\;}$ , representing an effective single binding site, was defined as the average of the fitted component means. This value served as a reference for quantification. The number of proteins per cluster was determined by calculating the ratio between each cluster’s qPAINT index and $\overset{®}{\mu_{1}\;}$ (see Supporting Information for details). Representative examples of single- and double-antibody-labeled Nup96 pairs identified by qPAINT analysis are shown in Figure 3b-c (left panels) for both OAR4- and OAR1-labeled NPCs, respectively.

It is important to note that all qPAINT measurements were performed in imaging buffer lacking the Trolox-based photostabilization system used for high-resolution DNA-PAINT imaging^32^ (Figure 2). Trolox is known to alter the binding kinetics of DNA-PAINT imager strands and is therefore incompatible with quantitative kinetic analysis. As a consequence, the effective spatial resolution of the qPAINT data sets is slightly reduced relative to those obtained under optimized DNA-PAINT imaging conditions (see CRLB and NeNA parameters, Table S1). This reduced resolution limits the reliable spatial separation of all individual Nup96 dimers within each NPC and explains why some symmetry-related positions cannot be unambiguously distinguished based on localization density alone. Importantly, however, qPAINT analysis relies on the temporal statistics of imager binding events rather than on complete spatial separation of adjacent structures. As long as binding events can be assigned to defined regions of interest, the extracted dark-time distributions remain proportional to the number of accessible binding sites. Thus, even under conditions of reduced spatial resolution, qPAINT retains its capacity to report biding-site numbers with high accuracy.

Consistent behavior was observed when labeling with the commercially available nanobody-DNA conjugate, in which the DNA-to-nanobody ratio is strictly one. The corresponding qPAINT histograms exhibited similarly narrow distributions and clear separation between singly and doubly labeled Nup96 pairs, comparable to those obtained with OAR1 and OAR4 conjugates (Figure S9, Figure 3b-c). Across nanobody, OAR1, and OAR4 labeling conditions, distributions of localization rates, clustering, and qPAINT-derived binding sites were highly consistent (Figure S10a–d), with similar fractions of monomeric and dimeric Nup96 pairs, indicating comparable overall labeling efficiencies. Quantification based on the number of Nup96 proteins per NPC determined by qPAINT (Figure S10e, Table S3) yielded effective labeling efficiencies of 40% for the nanobody, 50% for both OAR1 and OAR4. These values, which are in good agreement with previously reported^28^ ones for the GFP-1H1 nanobody, fall within a narrow range and demonstrate that the site-selectively conjugated full-length antibodies label Nup96–GFP with efficiencies comparable to the nanobody probe.

In contrast, NPCs labeled using antibodies conjugated using conventional lysine-based conjugation exhibited markedly broadened qPAINT index distributions (Figure 3d). The histogram extended toward higher qPAINT indices, indicating a fraction of dimers with abnormally high apparent docking-site numbers. Such behavior is consistent with the intrinsic chemical heterogeneity of amine-reactive labeling, which produces antibody populations carrying a variable and uncontrolled number of DNA strands, potentially ranging from one to four per antibody. Representative examples of individual NPCs (Figure 3d, left panel) demonstrate qPAINT-based classification of Nup96 pairs not only as monomeric and dimeric, but also as higher apparent labeling states (i.e., trimers, tetramers, etc.). Given that the stoichiometry of Nup96 within the NPC is structurally fixed and does not exceed two copies per symmetry-related position, these elevated qPAINT indices cannot reflect additional protein copies. Instead, they most plausibly arise from multiple DNA docking strands attached to a single antibody molecule, thereby inflating the kinetic readout. Under these conditions, the proportionality between qPAINT index and true protein copy number is disrupted. As a result, the kinetic signal becomes partially decoupled from the underlying biological stoichiometry, leading to systematic overestimation and reduced quantitative precision.

In summary, this work establishes site-selective disulfide rebridging with pyridazinedione chemistry as a robust strategy for producing stoichiometrically defined antibody–DNA conjugates. Both OAR1 and OAR4 constructs yield near homogeneous probes that preserve the proportionality between qPAINT index and protein copy number, enabling precise molecular counting even under conditions that limit spatial resolution. While OAR1 provides maximal conceptual clarity by enforcing a one-to-one DNA-to-antibody ratio, the practical considerations of probe preparation differ between the two approaches. The OAR4 strategy relies on dibromopyridazinedione (diBrPD) reagents, which are comparatively straightforward to synthesize and are now commercially available from various venders, making this route readily accessible to nonspecialist laboratories. By contrast, the tetra-PD scaffold required for OAR1 conjugation is not currently commercially available, as yet, and thus requires some chemical expertise. Nonetheless, once obtained, the OAR1 conjugation proceeds in a particularly clean and well-defined manner, yielding highly homogeneous products with minimal optimization.

Beyond the choice of OAR1 or OAR4, a key advantage of these site-selective strategies is their broad applicability to virtually any IgG antibody, providing a versatile platform for quantitative DNA-PAINT imaging of endogenous targets. While recombinant nanobodies and single-domain antibodies (sdAbs) can be engineered to achieve defined 1:1 DNA-to-protein stoichiometry and offer a reduced steric footprint due to their small size, this typically requires recombinant modification of the binding scaffold. Site-specific conjugation is commonly implemented through strategies such as the introduction of engineered cysteine residues for maleimide coupling or enzymatic approaches including sortase-mediated ligation.^33^ Although highly effective, these methods require redesign and recombinant production of each binder, which limits their straightforward integration into standard immunolabeling workflows and restricts their availability to a subset of targets for which suitable engineered binders are available. In contrast, the OAR1 and OAR4 approaches extend precise molecular counting to full-length antibodies, enabling accurate, reproducible, and multiplexed imaging across diverse protein targets and biological systems. Using the nuclear pore complex as a benchmark, we demonstrate that these well-defined conjugates preserve structural fidelity while supporting high-precision qPAINT measurements, establishing a practical and broadly accessible foundation for robust, quantitative super-resolution imaging.

Taken together, these findings establish PD-based antibody–DNA conjugation as a broadly applicable and practical solution to the limitations of conventional labeling strategies. By delivering homogeneous, stoichiometrically defined probes without compromising imaging performance, this approach enables accurate, reproducible molecular counting across diverse endogenous targets and complex cellular assemblies, providing a solid foundation for truly quantitative, multiplexed super-resolution studies.

## Supplementary Material



## Notes

Materials and methods, detailed organic synthesis and characterization data, information on the optical setup, cell culture and sample preparation procedures, DNA-PAINT docking and imager sequences, DNA-PAINT imaging experiments and data processing, and qPAINT analysis (PDF)
