# Supplementary material for: Stoichiometrically Defined Antibody–DNA Conjugates for Quantitative Super-Resolution Imaging
Source: Nano Lett. 2026 May 20;26(21):7166–73. doi: 10.1021/acs.nanolett.6c02173 (PMC13237764; doi:10.1021/acs.nanolett.6c02173)
Supplement: Supplementary file 1 [file nl6c02173_si_001.pdf]

Supporting Information

Stoichiometrically Defined Antibody–DNA Conjugates for Quantitative Super-Resolution Imaging

Luciana P. Martinez[1‡], Clíona McMahon[2‡], Cecilia Zaza[1], Olivia P. L. Dalby[1,2], Callum J. Stack[2], James R. Baker[2], Vijay Chudasama[2], and Sabrina Simoncelli[1,2*]*

1. London Centre for Nanotechnology, University College London, 19 Gordon Street, WC1H 0AH London, United Kingdom.
2. Department of Chemistry, University College London, 20 Gordon Street, WC1H 0AJ London, United Kingdom.

‡ These authors contributed equally to this work
* Email: s.simoncelli@ucl.ac.uk; v.chudasama@ucl.ac.uk;

Materials

All reagents were purchased from Sigma Aldrich, Alfa Aesar, ThermoFisher Scientific, BroadPharm or Biomers.net and were used as received unless otherwise stated. Anti-GFP antibody refers to AB02257-10.3-BT Anti-GFP (N86/38.1).

NMR Spectroscopy

Nuclear magnetic resonance (NMR) spectra were recorded on Bruker Avance Neo 700, Avance III 600, DRX 500, or Avance III 400 spectrometers at 25 °C. Chemical shifts (δ) are reported in parts per million (ppm) relative to tetramethylsilane (TMS), calibrated using the residual solvent signals. In the case of amide rotamers, and where applicable, only the major rotamer has been assigned; integrations reflect the combined areas of all rotameric peaks. Coupling constants (J) are reported in Hertz (Hz) and correspond to $3J_{H\text{–}H}$ couplings between protons. Infrared (IR) spectra were recorded on a PerkinElmer Spectrum 100 FTIR spectrometer operating in ATR mode. Small-molecule mass spectra were acquired by the UCL Mass Spectrometry Facility using an Orbitrap Q Exactive mass spectrometer.

Organic Synthesis

Di-*tert*-butyl-1-methylhydrazine-1,2-dicarboxylate

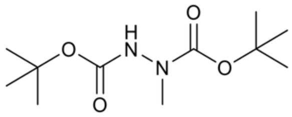

The following procedure was adapted from work by Bahou et al.[1] To a solution of 1-boc-1-methylhydrazine (3.00 mL, 20.2 mmol) in i-PrOH (16 mL) was added dropwise over 30 min di-*tert*-butyl dicarbonate (5.00 g, 22.9 mmol) pre-dissolved in DCM (12 mL). The reaction mixture was stirred at 21 °C for 18 h. Following this, the solvents were removed *in vacuo* and the crude residue was purified by flash column chromatography (0% to 20% EtOAc/cyclohexane) to afford di-*tert*-butyl-1-methylhydrazine1,2-dicarboxylate (4.29 g, 17.4 mmol, 86%) as a white solid.

^{1}H NMR (600 MHz, $CDCl_3$, major rotamer) δ 6.40 (s, 1H) 3.10 (s, 3H), 1.46 (s 18H).

^{13}C NMR (150 MHz, $CDCl_3$, major rotamer) δ 155.9 (C), 81.1 (C), 37.6 (CH_3), 28.3 (CH_3).

IR (solid) 3314, 2980, 2933, 1702 cm^{-1}.

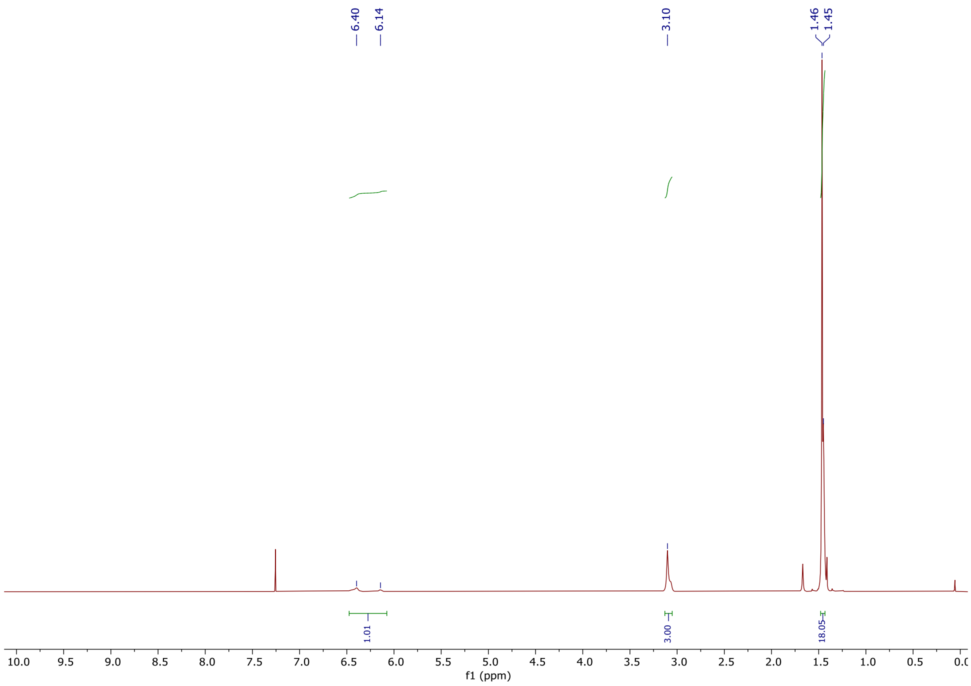
6.40
6.14
3.10
1.46
1.45
1.01
3.00
18.05
10.0
9.5
9.0
8.5
8.0
7.5
7.0
6.5
6.0
5.5
5.0
4.5
4.0
3.5
3.0
2.5
2.0
1.5
1.0
0.5
f1 (ppm)

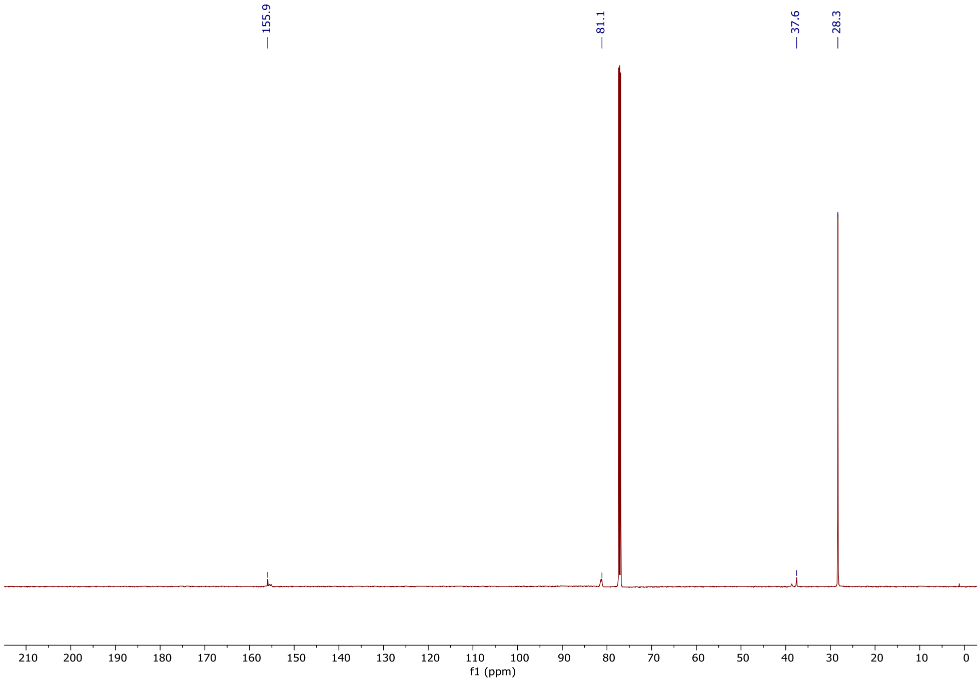
155.9
81.1
37.6
28.3
210
200
190
180
170
160
150
140
130
120
110
100
90
80
70
60
50
40
30
20
10
0
f1 (ppm)

Di-*tert*-butyl-1-(3-(*tert*-butoxy)-3-oxopropyl)-2-methylhydrazine-1,2-dicarboxylate [1]

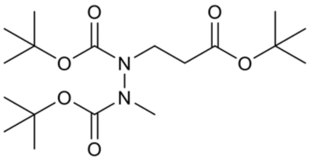

To a solution of di-*tert*-butyl 1-methylhydrazine-1,2-dicarboxylate (3.75 g, 15.2 mmol) in *tert*-butanol (25 mL) was added 2 M aqueous NaOH (0.5 mL), and the reaction mixture was stirred at 21 °C for 10 min. After this, *tert*-butyl acrylate (6.63 mL, 45.7 mmol) was then added, and the reaction mixture was heated under reflux for 68 h. The solvent was removed *in vacuo*, and the residue was suspended in water (50 mL). The product was then extracted into ethyl acetate (8 × 50 mL) and dried ($MgSO_4$) to give di-*tert*-butyl-1-(3-(*tert*-butoxy)-3-oxopropyl)-2-methylhydrazine-1,2-dicarboxylate as a clear oil (4.95 g, 13.2 mmol, 87%).
^{1}H NMR (600 MHz, $CDCl_3$, rotamers) δ 3.84–3.51 (m, 2H), 3.05–2.98 (m, 3H), 2.52-2.49 (m, 2H), 1.47–1.42 (m, 27H).
^{13}C NMR (150 MHz, $CDCl_3$, rotamers) δ 171.1 (C), 155.5 (C), 154.5 (C), 81.1 (C), 80.9 (C), 44.6 (CH_2), 36.7 (CH_3), 34.2 (CH_2), 28.4 (CH_3), 28.2 (CH_3).
IR (thin film) 2977, 2933, 1709 cm^{-1}.

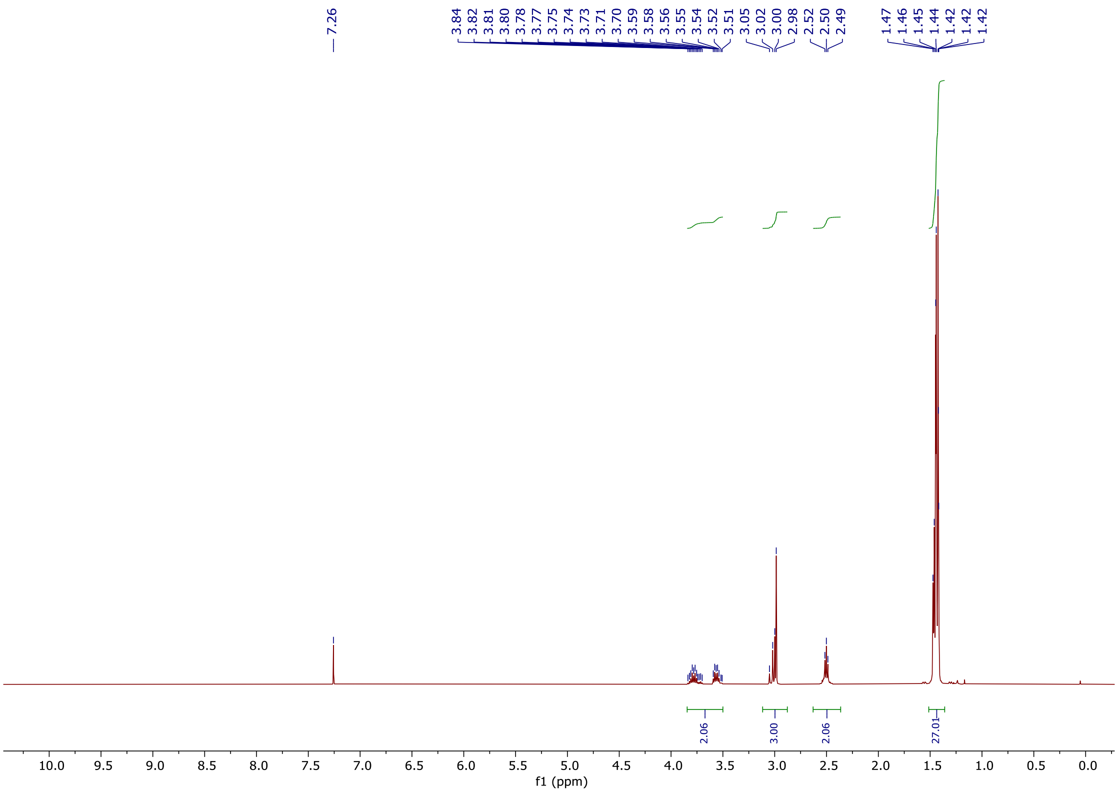
7.26
3.84
3.82
3.81
3.80
3.78
3.77
3.75
3.74
3.73
3.71
3.70
3.59
3.58
3.56
3.55
3.54
3.52
3.51
3.05
3.02
3.00
2.98
2.52
2.50
2.49
1.47
1.46
1.45
1.44
1.42
1.42
1.42
2.06
3.00
2.06
27.01
10.0 9.5 9.0 8.5 8.0 7.5 7.0 6.5 6.0 5.5 5.0 4.5 4.0 3.5 3.0 2.5 2.0 1.5 1.0 0.5 0.0
f1 (ppm)

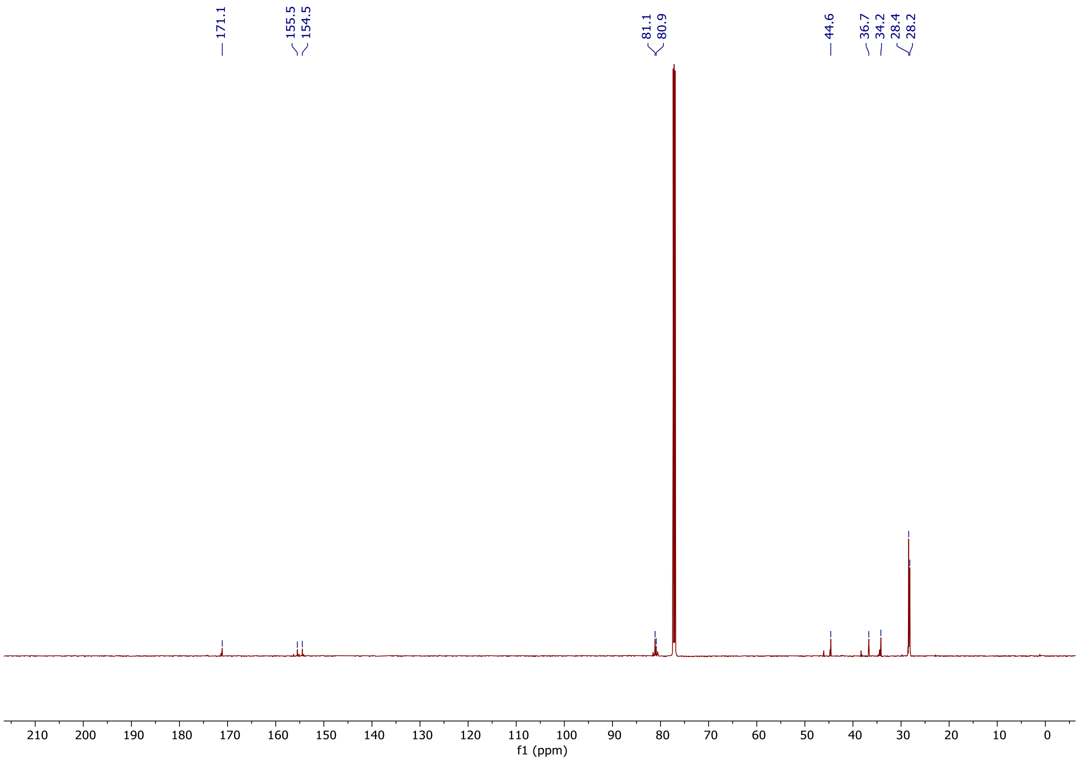
171.1
155.5
154.5
81.1
80.9
44.6
36.7
34.2
28.4
28.2
210 200 190 180 170 160 150 140 130 120 110 100 90 80 70 60 50 40 30 20 10 0
f1 (ppm)

3-(4,5-Dibromo-2-methyl-3,6-dioxo-3,6-dihydropyridazin-1(2*H*)-yl) propanoic acid (MetPAc) [1]

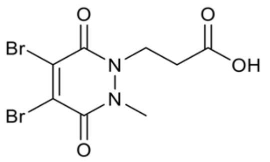

Dibromomaleic acid Error! Reference source not found.(5.32 g, 19.4 mmol) was dissolved in AcOH (100 mL), and the solution was heated under reflux for 30 min. di-*tert*-butyl-1-(3-(*tert*-butoxy)-3-oxopropyl)-2-methylhydrazine-1,2-dicarboxylate (6.08 g, 16.2 mmol) was then added, and the reaction mixture was heated under reflux for a further 6 h. The reaction mixture was then concentrated *in vacuo* and co-evaporated with toluene (3 × 30 mL, as an azeotrope) and chloroform (30 mL). The crude residue was purified by flash column chromatography (50% to 100% EtOAc/cyclohexane (1% AcOH)) to afford 3-(4,5-dibromo-2-methyl-3,6-dioxo-3,6-dihydropyridazin-1(2*H*)-yl) propanoic acid (4.66 g, 13.1 mmol, 81%) as a yellow solid.

^{1}H NMR (600 MHz, MeOD) δ 4.43 (t, J = 7.3 Hz, 2H), 3.69 (s, 3H), 2.74 (t, J = 7.3 Hz, 2H).

^{13}C NMR (150 MHz, MeOD) δ 173.8 (C), 154.7 (C), 154.5 (C), 136.7 (C), 136.4 (C), 44.9 (CH_3), 35.4 (CH_2), 32.5 (CH_2).

IR (thin film) 3226, 2945, 2835, 1731, 1660, 1572 cm^{-1}.

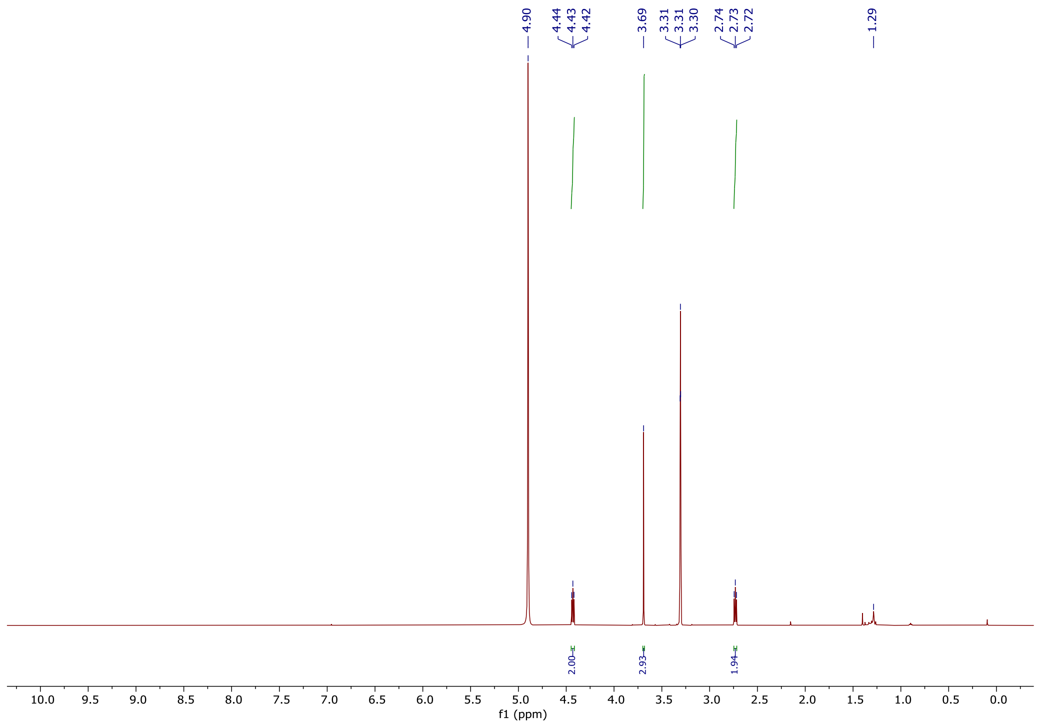
4.90
4.44
4.43
4.42
3.69
3.31
3.31
3.30
2.74
2.73
2.72
1.29
2.00
2.93
1.94
10.0 9.5 9.0 8.5 8.0 7.5 7.0 6.5 6.0 5.5 5.0 4.5 4.0 3.5 3.0 2.5 2.0 1.5 1.0 0.5 0.0
f1 (ppm)

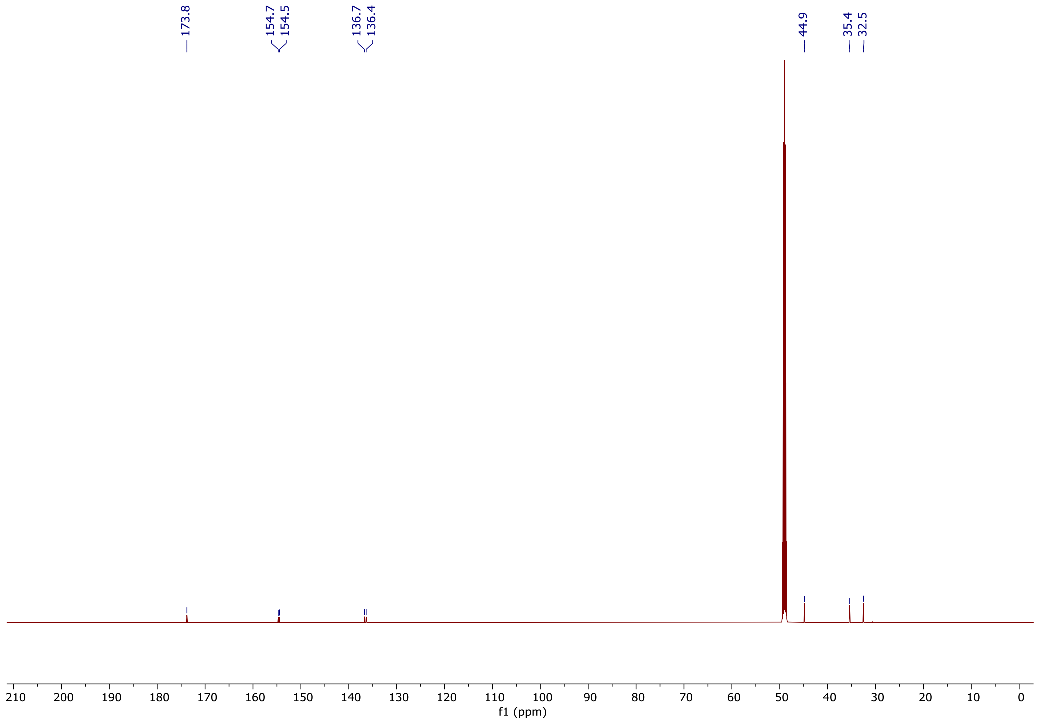
173.8
154.7
154.5
136.7
136.4
44.9
35.4
32.5
210 200 190 180 170 160 150 140 130 120 110 100 90 80 70 60 50 40 30 20 10 0
f1 (ppm)

2,5-Dioxopyrrolidin-1-yl 3-(4,5-dibromo-2-methyl-3,6-dioxo-3,6-dihydropyridazin1(2*H*)-yl) propanoate [1]

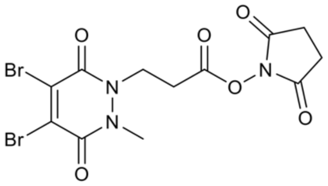

To a solution of 3-(4,5-dibromo-2-methyl-3,6-dioxo-3,6-dihydropyridazin-1(2*H*)-yl)propanoic acid (704 mg, 1.98 mmol) in THF (20 mL), pre-cooled to 0 °C, was added *N*-(3-dimethylaminopropyl)-*N*'-ethylcarbodiimide (414 mg, 2.16 mmol). The homogeneous solution was then stirred at 0 °C for 30 min, after which *N*-hydroxysuccinimide (249 mg, 2.16 mmol) was added and the reaction mixture was stirred at 21 °C for a further 18 h. The solvent was then removed *in vacuo*, and the crude residue was dissolved in DCM. The resulting suspension was filtered, and the filtrate was concentrated *in vacuo*. Purification of the crude residue by flash column chromatography (50% to 100% EtOAc/cyclohexane) afforded 2,5-dioxopyrrolidin-1-yl 3-(4,5-dibromo-2-methyl-3,6-dioxo-3,6-dihydropyridazin-1(2*H*)-yl) propanoate (488 mg, 0.99 mmol, 50%) as a yellow solid.

^{1}H NMR (600 MHz, $CDCl_3$) δ 4.47 (t, *J* = 6.9 Hz, 2H), 3.67 (s, 3H), 3.10 (t, *J* = 6.9 Hz, 2H), 2.84 (s, 4H).

^{13}C NMR (151 MHz, $CDCl_3$) δ 168.8 (C), 166.1 (C), 153.3 (C), 153.1 (C), 136.9 (C), 135.3 (C), 43.0 (CH_2), 35.3 (CH_3), 29.1 (CH_2), 25.7 (CH_2).

IR (solid) 2919, 2852, 1810, 1780, 1732, 1624, 1577 cm^{-1}.

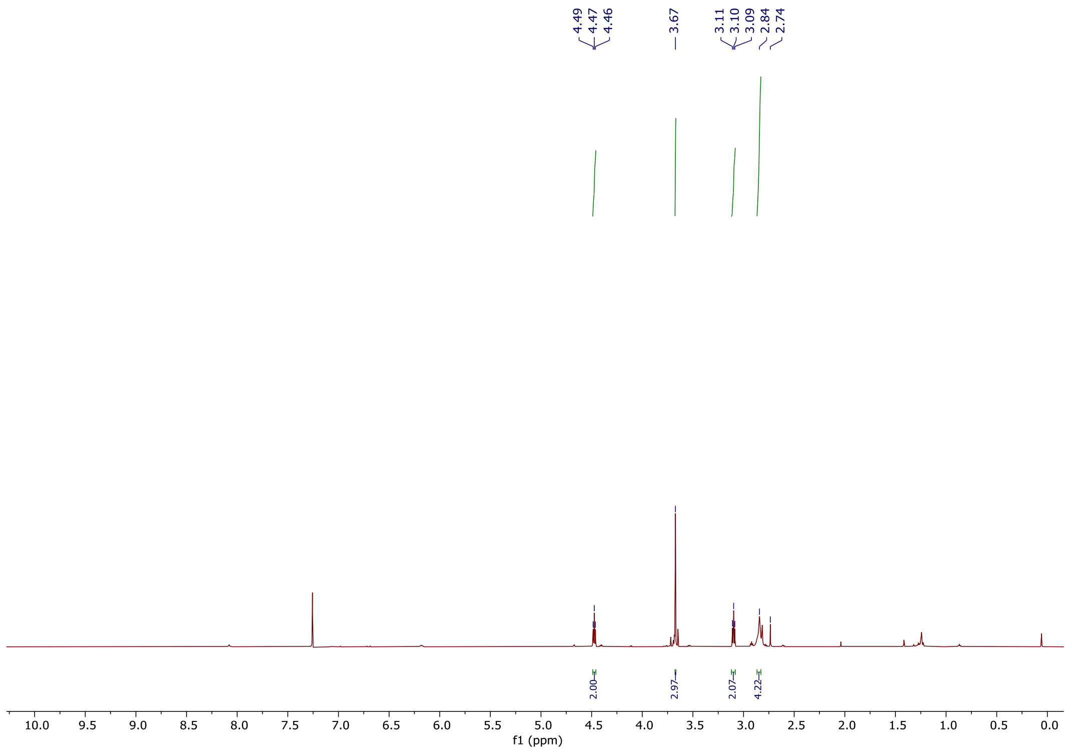
4.49
4.47
4.46
3.67
3.11
3.10
3.09
2.84
2.74
2.00
2.97
2.07
4.22
f1 (ppm)

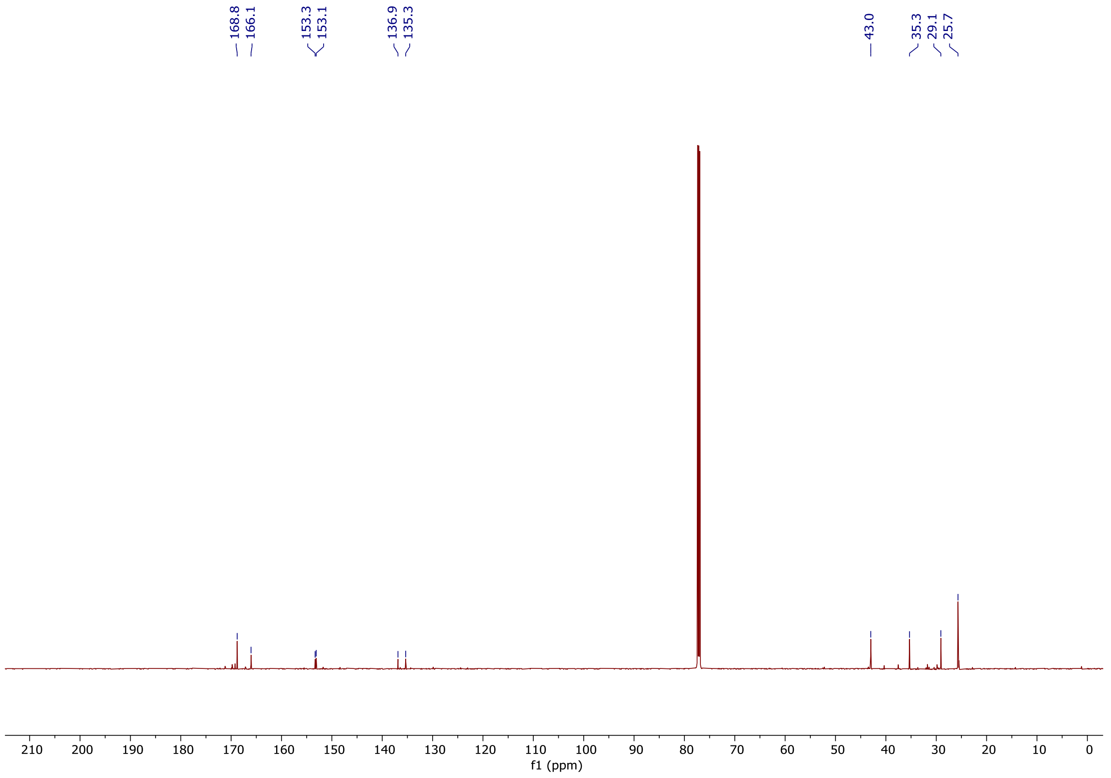
168.8
166.1
153.3
153.1
136.9
135.3
43.0
35.3
29.1
25.7
f1 (ppm)

((1*R*,8*S*,9*s*)-Bicyclo[6.1.0]non-4-yn-9-yl)methyl (2-(2-(2-(3-(4,5-dibromo-2-methyl-3,6-dioxo-3,6-dihydropyridazin-1(2*H*)-yl)propanamido)ethoxy)ethoxy)ethyl)carbamate (BCN PD) [1]

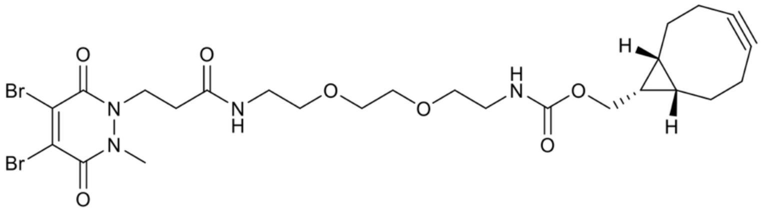

To a solution of 2,5-dioxopyrrolidin-1-yl 3-(4,5-dibromo-2-methyl-3,6-dioxo-3,6-dihydropyridazin-1(2*H*)-yl) propanoate (70 mg, 0.15 mmol) in dry MeCN (10 mL) was added *N*-[(1*R*,8*S*,9*s*)-bicyclo[6.1.0]non-4-yn-9-ylmethyloxycarbonyl]-1,8-diamino-3,6-dioxaoctane (50 mg, 0.15 mmol), and the reaction stirred at 21 °C for 18 h. After this time, the solvent was removed *in vacuo* and the crude residue dissolved in $CHCl_3$ (50 mL). The organic solution was then washed with water (3 × 30 mL), followed by saturated aq. K_2CO_3 (30 mL). The organic layer was then dried ($MgSO_4$) and concentrated *in vacuo.* Purification of the crude residue by flash column chromatography (0% to 10% MeOH/EtOAc) afforded ((1*R*,8*S*,9*s*)-bicyclo[6.1.0]non-4-yn-9-yl)methyl (2-(2-(2-(3-(4,5-dibromo-2-methyl-3,6-dioxo-3,6-dihydropyridazin-1(2*H*)-yl)propanamido)ethoxy)ethoxy)ethyl) carbamate (51.2 mg, 0.08 mmol, 51%) as a yellow gum.

^{1}H NMR (700 MHz, $CDCl_3$, rotamers) δ 7.78 (s, 0.2H*), 6.30 (s, 0.6H*), 5.74 (s, 0.2H*), 5.21 (s, 0.6H*), 4.44 (t, *J* = 7.0 Hz, 2H), 4.15-4.14 (m, 2H), 3.72 (s, 3H), 3.64–3.36 (m, 12H), 2.62 (t, *J* = 6.9 Hz, 2H), 2.33–2.19 (m, 6H), 1.61–1.57** (m, 2H), 1.43–1.26 (m, 3H), 0.95 (d, *J* = 9.0 Hz, 2H).

^{13}C NMR (176 MHz, $CDCl_3$, rotamers) δ 169.0 (C), 157.0 (C), 153.1 (C), 153.0 (C), 136.4 (C), 135.5 (C), 98.9 (C), 70.4 (CH_2), 70.3 (CH_2), 69.7 (CH_2), 63.0 (CH_2), 44.6 (CH_2), 40.9 (CH_2), 39.5 (CH_2), 35.1 (CH_3), 34.1 (CH_2), 29.2 (CH_2), 21.6 (CH_2), 20.3 (CH_2), 17.9 (CH), 14.2 (CH).

IR (thin film) 3329, 2920, 2858, 1708, 1630, 1572, 1534 cm^{-1}.

* Less than 1H potentially due to partial D-H exchange of the acidic NH amide proton in the NMR sample.

**Behind water peak

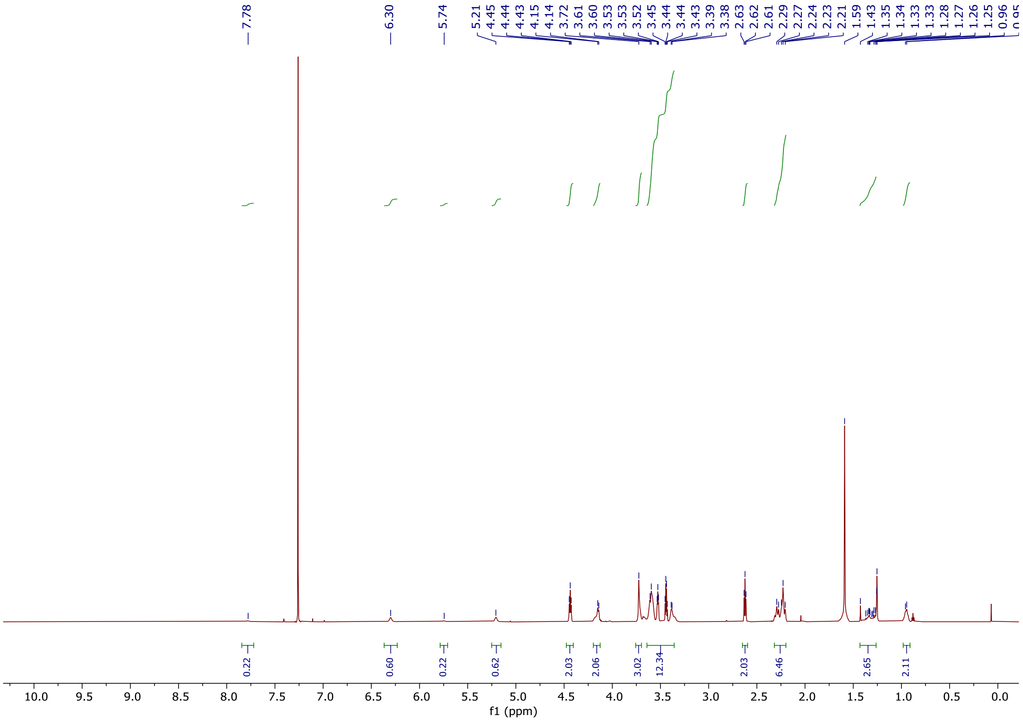

7.78
6.30
5.74
5.21
4.45
4.44
4.43
4.15
4.14
3.72
3.61
3.60
3.53
3.53
3.52
3.45
3.44
3.44
3.43
3.39
3.38
2.63
2.62
2.61
2.29
2.27
2.24
2.23
2.21
1.59
1.43
1.35
1.34
1.33
1.33
1.28
1.27
1.26
1.25
0.96
0.22
0.60
0.22
0.62
2.03
2.06
3.02
12.34
2.03
6.46
2.65
2.11
10.0 9.5 9.0 8.5 8.0 7.5 7.0 6.5 6.0 5.5 5.0 4.5 4.0 3.5 3.0 2.5 2.0 1.5 1.0 0.5 0.0
f1 (ppm)

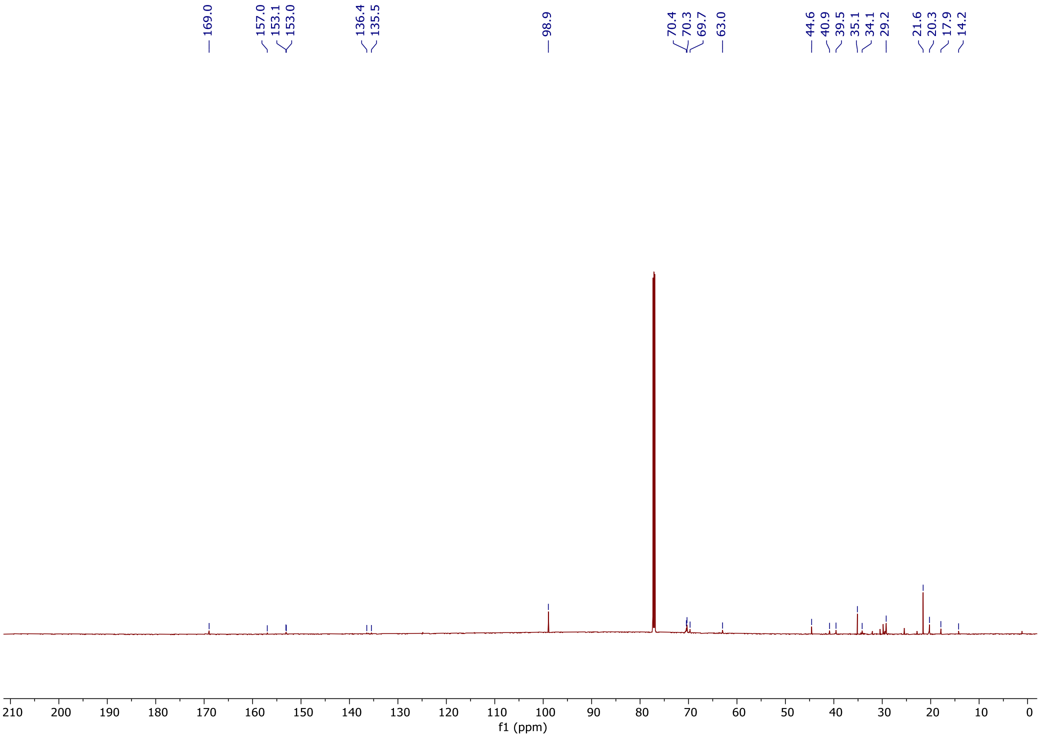

169.0
157.0
153.1
153.0
136.4
135.5
98.9
70.4
70.3
69.7
63.0
44.6
40.9
39.5
35.1
34.1
29.2
21.6
20.3
17.9
14.2
210 200 190 180 170 160 150 140 130 120 110 100 90 80 70 60 50 40 30 20 10 0
f1 (ppm)

Di-*tert*-butyl (azanediylbis(ethane-2,1-diyl))dicarbamate [2]

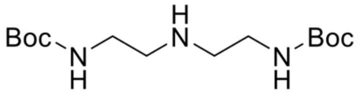

The following procedure was adapted from work by Dannheim et al.[2] To a solution of diethylenetriamine (1.08 mL, 10.0 mmol) in THF (12 mL) at 0 °C was slowly added a solution of Boc-ON (4.96 g, 20.0 mmol) pre-dissolved in THF (12 mL). The reaction was stirred at 0 °C under an inert atmosphere for 1.5 h. After this time, the solvent was removed *in vacuo* and the crude product dissolved in DCM (50 mL). The product was washed with 2 M NaOH (50 mL), then extracted from the aqueous phase with DCM (50 mL). The organic layers were combined, washed with water (50 mL), brine (50 mL) and dried ($MgSO_4$) to yield di-*tert*-butyl (azanediylbis(ethane-2,1-diyl))dicarbamate (3.00 g, 10.0 mmol, 100%) as a white solid.

^{1}H NMR (600 MHz, $CDCl_3$) δ 4.95 (s, 2H), 3.20 (q, *J* = 6.1 Hz, 4H), 2.71 (t, *J* = 5.8 Hz, 4H), 1.43 (s, 18H).

^{13}C NMR (151 MHz, $CDCl_3$) δ 156.4 (C), 79.4 (C), 48.9 (CH_2), 40.0 (CH_2), 28.5(CH_3).

IR (solid) 3357, 2944, 2926, 2779, 2707, 2663, 2484, 2457, 1696, 1529 cm^{-1}.

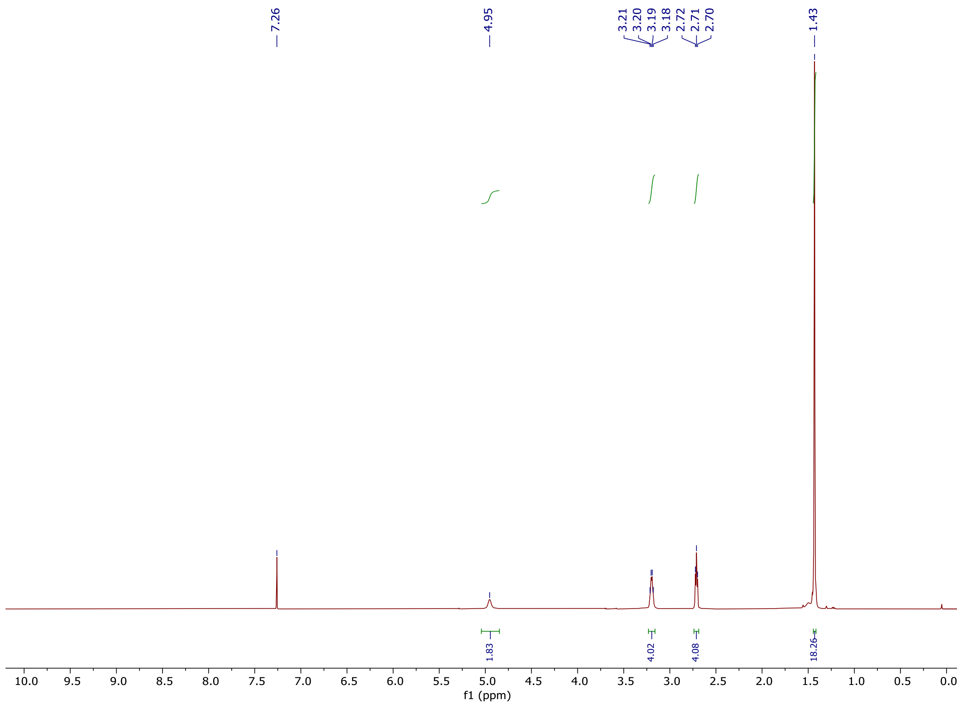
7.26
4.95
3.21
3.20
3.19
3.18
2.72
2.71
2.70
1.43
1.83
4.02
4.08
18.26
10.0 9.5 9.0 8.5 8.0 7.5 7.0 6.5 6.0 5.5 5.0 4.5 4.0 3.5 3.0 2.5 2.0 1.5 1.0 0.5 0.0
f1 (ppm)

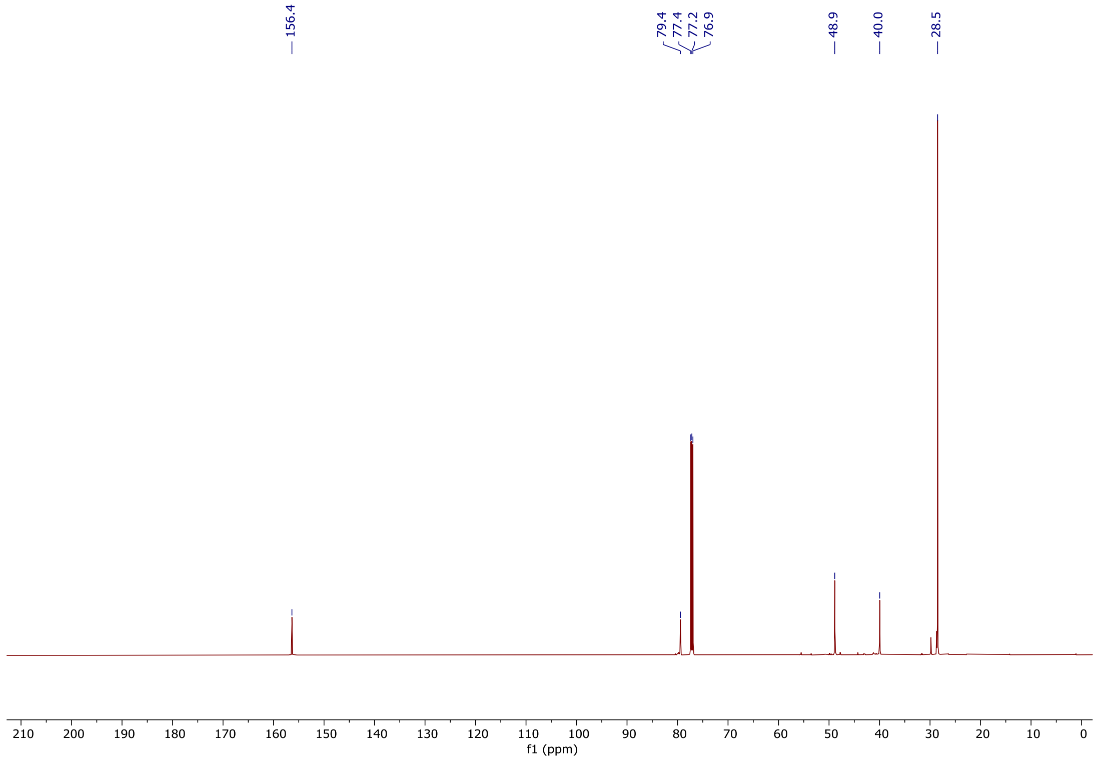
156.4
79.4
77.4
77.2
76.9
48.9
40.0
28.5
210 200 190 180 170 160 150 140 130 120 110 100 90 80 70 60 50 40 30 20 10 0
f1 (ppm)

Methyl bis(2-((*tert*-butoxycarbonyl)amino)ethyl)glycinate [2]

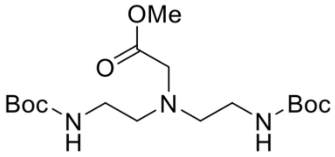

To a solution of di-*tert*-butyl (azanediylbis(ethane-2,1-diyl))dicarbamate (3.00 g, 10.0 mmol) in DMF (30 mL) was added DIPEA (2.14 mL, 12.0 mmol) and methyl bromoacetate (1.42 mL, 15.0 mmol). The reaction was stirred at room temperature for 16 h. After this time the solvent was removed *in vacuo* and the crude product dissolved in EtOAc (70 mL). The product was washed with water (2 × 50 mL), LiCl (20 mL) and brine (20 mL) then dried ($MgSO_4$). The solvent was removed *in vacuo* to give the methyl bis(2-((*tert*-butoxycarbonyl)amino)ethyl)glycinate (3.76 g, 10.0 mmol, 100%) as a colourless oil.
^{1}H NMR (600 MHz, $CDCl_3$) δ 5.16 (s, 2H), 3.70 (s, 3H), 3.37 (s, 2H), 3.15 (q, *J* = 5.7 Hz, 4H), 2.71 (t, *J* = 5.8 Hz, 4H), 1.44 (s, 18H).
^{13}C NMR (151 MHz, $CDCl_3$) δ 172.3 (C), 156.3 (C), 79.3 (C), 55.0 (CH_2), 54.2 (CH_2), 51.8 (CH_2), 38.7 (CH_3), 28.6 (CH_3).
IR (thin film) 3358, 2976, 2928, 2853, 1688, 1509 cm^{-1}.

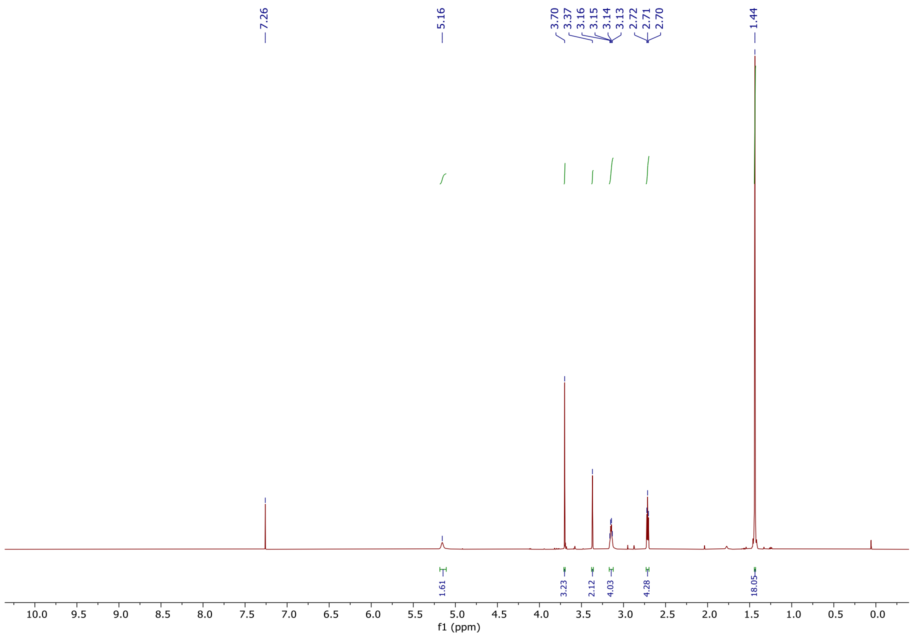

7.26
5.16
3.70
3.37
3.16
3.15
3.14
3.13
2.72
2.71
2.70
1.44
1.61
3.23
2.12
4.03
4.28
18.05
10.0 9.5 9.0 8.5 8.0 7.5 7.0 6.5 6.0 5.5 5.0 4.5 4.0 3.5 3.0 2.5 2.0 1.5 1.0 0.5 0.0
f1 (ppm)

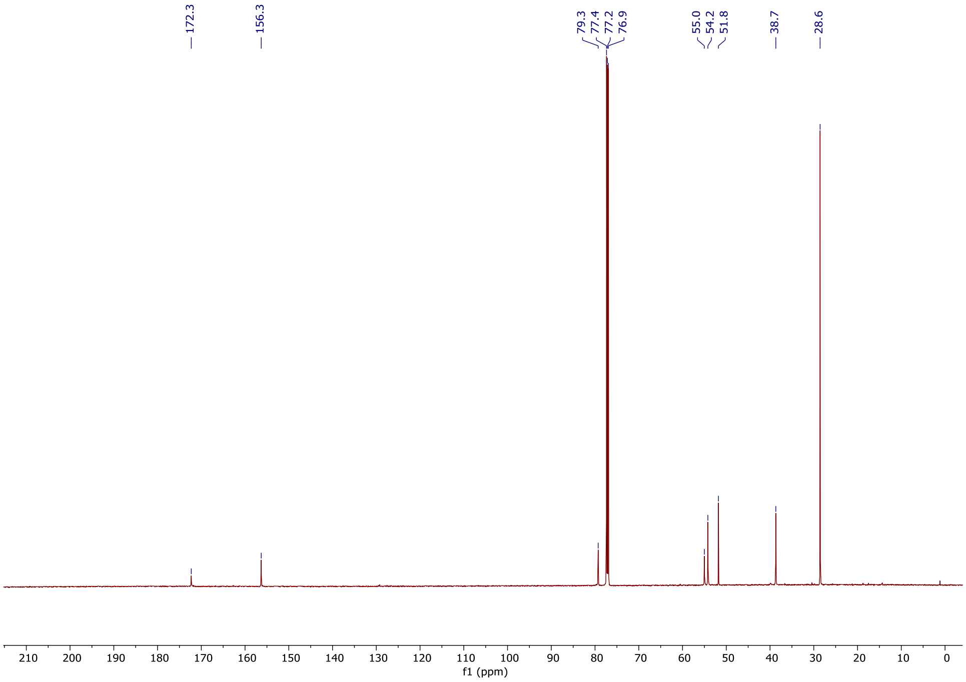

172.3
156.3
79.3
77.4
77.2
76.9
55.0
54.2
51.8
38.7
28.6
210 200 190 180 170 160 150 140 130 120 110 100 90 80 70 60 50 40 30 20 10 0
f1 (ppm)

Tetra Boc Amine Scaffold [2]

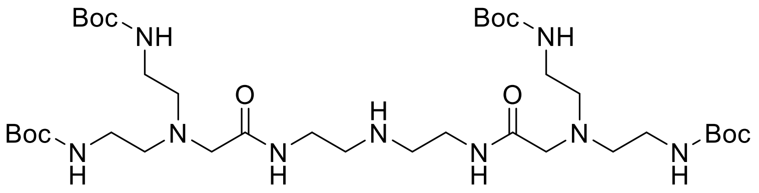

211

To a solution of methyl bis(2-((*tert*-butoxycarbonyl)amino)ethyl)glycinate (3.76 g, 10.0 mmol) in anh. MeOH (10 mL) was added DIPEA (892 μL, 5.0 mmol) and diethylenetriamine (270 μL, 2.5 mmol). The reaction was refluxed for 13 days. After this time, the solvent was removed *in vacuo* and the crude product purified *via* flash column chromatography (0-15% MeOH/DCM) to give tetra Boc amine scaffold (1.61 g, 2.0 mmol, 81%) as a white solid.

^{1}H NMR (500 MHz, MeOD) δ 3.39 (t, *J* = 6.2 Hz, 4H), 3.17 (s, 4H), 3.13 (t, *J* = 6.2 Hz, 8H), 2.84 (t, *J* = 6.3 Hz, 4H), 2.60 (t, *J* = 6.2 Hz, 8H), 1.45 (s, 36H).

^{13}C NMR (151 MHz, MeOD) δ 175.2 (C), 158.6 (C), 80.2 (C), 60.0 (CH_2), 56.4 (CH_2), 49.5 (CH_2), 39.7 (CH_2), 38.9 (CH_2), 28.9 (CH_3).

IR (thin film) 3370, 3297, 2977, 2934, 2827, 1683, 1654, 1519 cm^{-1}.

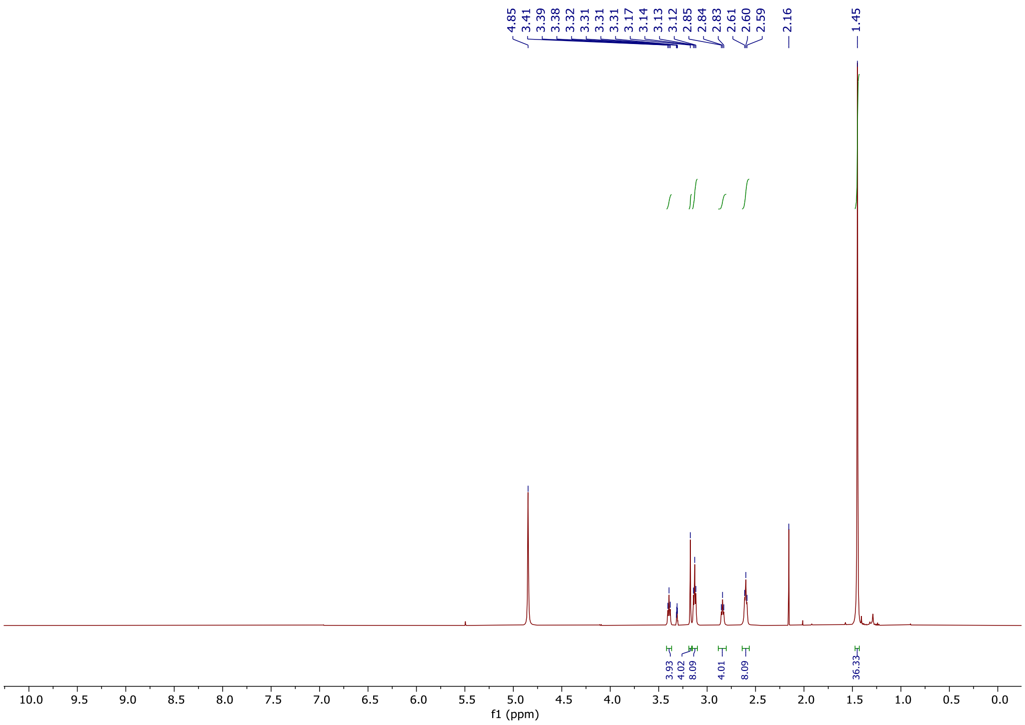
4.85
3.41
3.39
3.38
3.32
3.31
3.31
3.31
3.17
3.14
3.13
3.12
2.85
2.84
2.83
2.61
2.60
2.59
2.16
1.45
3.93
4.02
8.09
4.01
8.09
36.33
10.0 9.5 9.0 8.5 8.0 7.5 7.0 6.5 6.0 5.5 5.0 4.5 4.0 3.5 3.0 2.5 2.0 1.5 1.0 0.5 0.0
f1 (ppm)

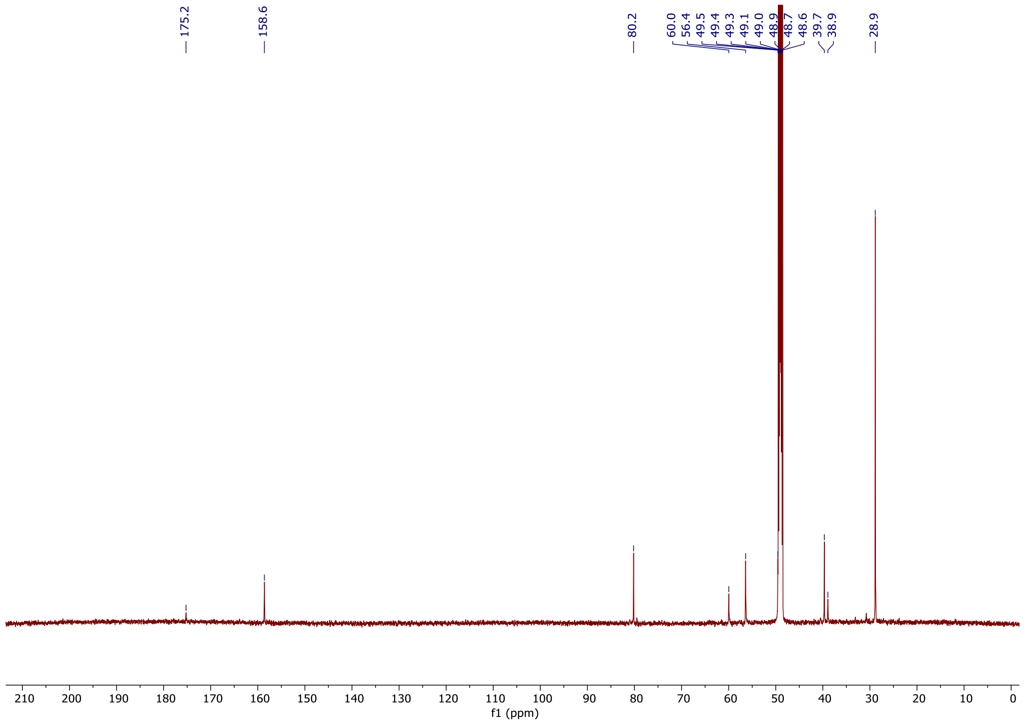
175.2
158.6
80.2
60.0
56.4
49.5
49.4
49.3
49.1
49.0
48.9
48.7
48.6
39.7
38.9
28.9
210 200 190 180 170 160 150 140 130 120 110 100 90 80 70 60 50 40 30 20 10 0
f1 (ppm)

N-(2-(2-(2-(2-azidoethoxy)ethoxy)ethoxy)ethyl)-2-(4-(6-methyl-1,2,4,5-tetrazin-3-yl)phenyl)acetamide

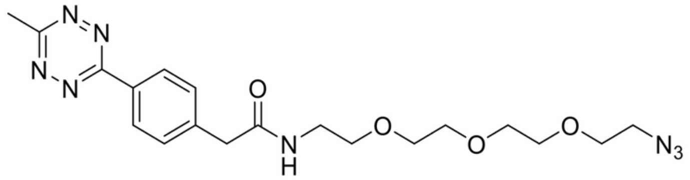

To a solution of methyltetrazine NHS ester (100 mg, 0.31 mmol) in DCM (4 mL) at 0 °C was added dropwise 11-azido-3,6,8-trioxaundecan-1-amine (90.9 µL, 0.46 mmol) in DCM (1 mL), pre-mixed with DIPEA (55 µL, 0.31 mmol). The reaction was allowed to warm to 22 °C and was stirred for 24 h. After this time, the reaction was diluted in DCM (30 mL) and washed with 4 M HCl (10 × 10 mL). The organic layer was dried ($MgSO_4$), the solvent removed *in vacuo* and the crude product purified *via* flash column chromatography (50-100% EtOAc/cyclohexane) to give *N*-(2-(2-(2-(2-azidoethoxy)ethoxy)ethoxy)ethyl)-2-(4-(6-methyl-1,2,4,5-tetrazin-3-yl)phenyl)acetamide (108 mg, 0.25 mmol, 81%) as a pink solid.

^{1}H NMR (600 MHz, $CDCl_3$) δ 8.56 (d, *J* = 8.3 Hz, 2H), 7.53 (d, *J* = 8.1 Hz, 2H), 3.69 – 3.64 (m, 8H), 3.60 (d, *J* = 5.4 Hz, 4H), 3.56 – 3.53 (m, 2H), 3.47 (q, *J* = 5.2 Hz, 2H), 3.38 (t, *J* = 5.0 Hz, 2H), 3.10 (s, 3H).

^{13}C NMR (151 MHz, $CDCl_3$) δ 170.3 (C), 167.4 (C), 164.0 (C), 140.2 (C), 130.4 (C), 128.4 (CH), 124.9 (CH), 70.8 (CH_2), 70.7 (CH_2), 70.7 (CH_2), 70.4 (CH_2), 70.2 (CH_2), 69.9 (CH_2), 50.9 (CH_2), 43.73 (CH_2), 39.6 (CH_2), 22.8 (CH_3).*Some hexane and grease present

IR (solid) 2954, 2917, 2849, 2105, 1738, 1660, 1612, 1546 cm^{-1}.

LRMS (ESI) 431 (100, $[M+H]^+$); **HRMS (ESI)** calcd for $C_{19}H_{27}O_4N_8[M+H]^+$ 431.2150; observed 431.2141.

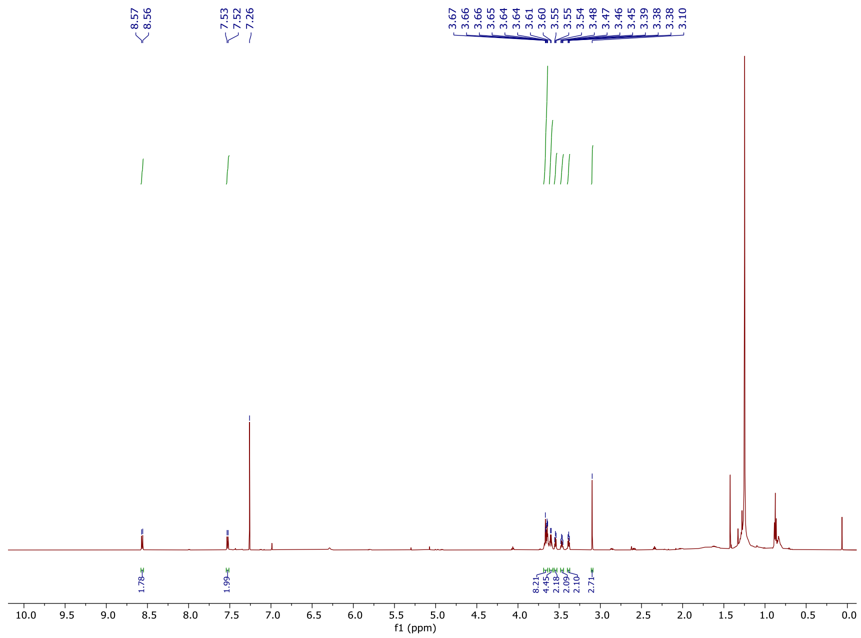
8.57
8.56
7.53
7.52
7.26
3.67
3.66
3.66
3.65
3.64
3.64
3.61
3.60
3.55
3.55
3.54
3.48
3.47
3.46
3.45
3.39
3.38
3.38
3.10
1.78
1.99
8.21
4.45
2.18
2.09
2.10
2.71
f1 (ppm)

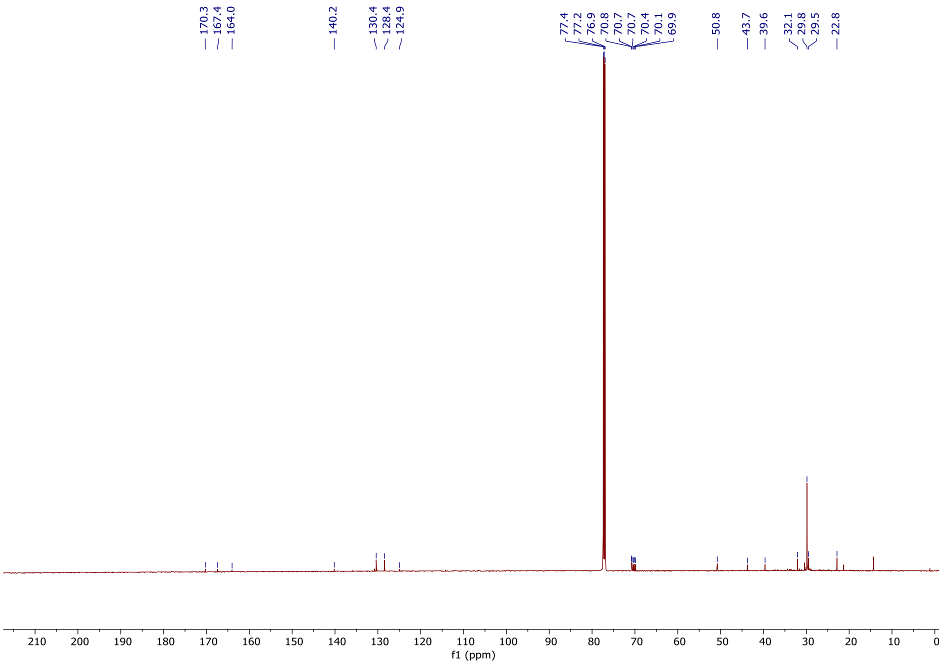
170.3
167.4
164.0
140.2
130.4
128.4
124.9
77.4
77.2
76.9
70.8
70.7
70.7
70.4
70.1
69.9
50.8
43.7
39.6
32.1
29.8
29.5
22.8
f1 (ppm)

2-Bromo-*N*-(prop-2-yn-1-yl)acetamide [2]

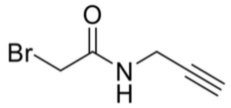

To a solution of propargyamine (0.58 mL, 9.1 mmol) in DCM (15 mL) at maximum stirring was added sat. $NaHCO_3$ (2.5 mL) and the solution cooled to -10 °C. To the reaction was added 2-bromoacetyl bromide (1.21 mL, 13.6 mmol) dropwise over 15 min. After this time, the reaction was allowed to warm to 22 °C. Upon completion the reaction mixture was concentration *in vacuo* and diluted with water (15 mL). The product was extracted with EtOAc (4 × 40 mL) and the combined organic phases washed with sat. $NaHCO_3$ (15 mL), 5% HCl (15 mL), brine (15 mL) and dried (Na_2SO_4). The solvent was removed *in vacuo* to give 2-bromo-*N*-(prop-2-yn-1-yl)acetamide (1.6 g, 1.9 mmol, 100%) as a yellow solid.

^{1}H NMR (500 MHz, $CDCl_3$) δ 6.65 (s, 1H), 4.09 (dd, *J* = 5.4, 2.5 Hz, 2H), 3.90 (s, 2H), 2.28 (t, *J* = 2.6 Hz, 1H).

^{13}C NMR (126 MHz, $CDCl_3$) δ 165.2 (C), 78.6 (C), 72.4 (CH), 30.1 (CH_2), 28.8 (CH_2).

IR (solid) 3286. 3229, 3046, 3006, 2956, 2919, 2850, 1726, 1668, 1634, 1533 cm^{-1}.

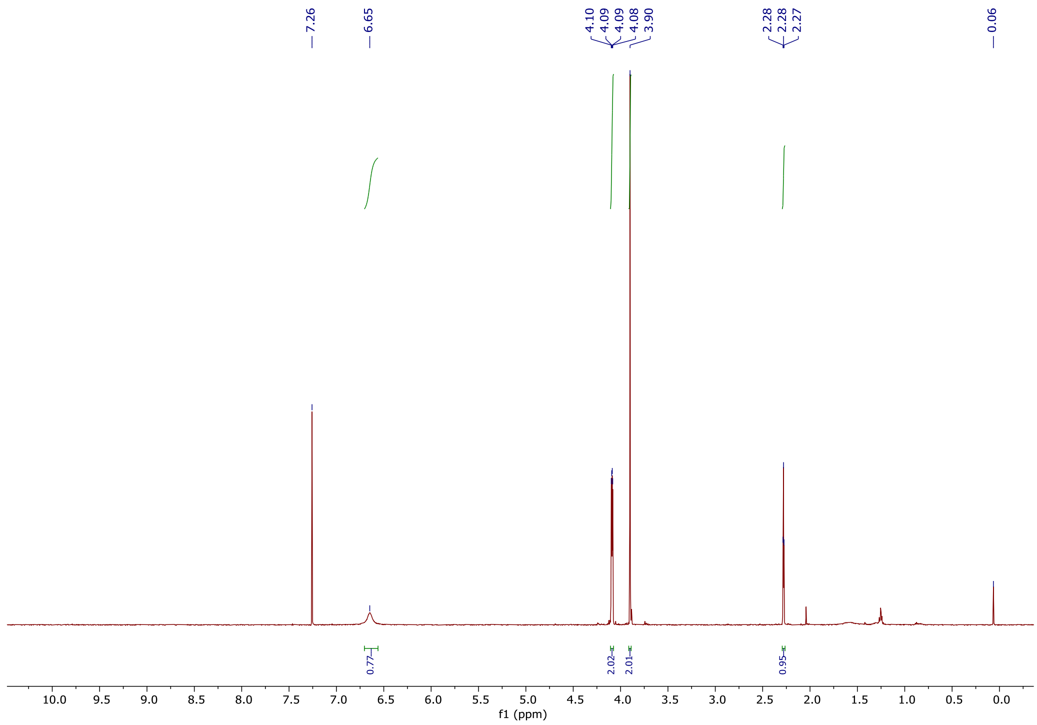
7.26
6.65
4.10
4.09
4.09
4.08
3.90
2.28
2.28
2.27
0.06
0.77
2.02
2.01
0.95
f1 (ppm)

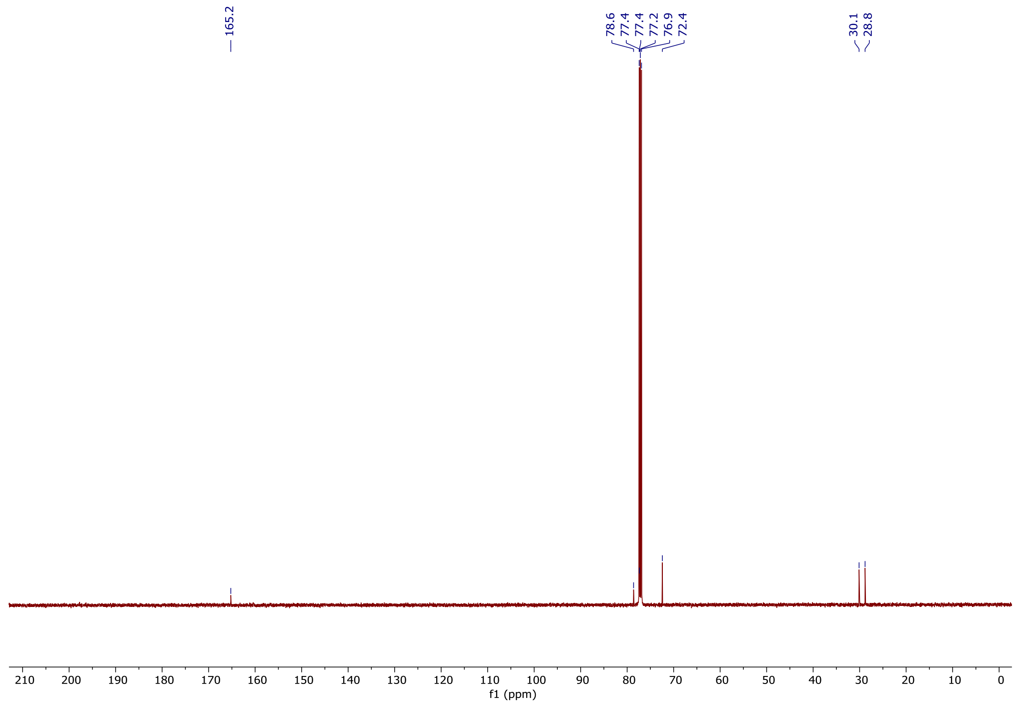
165.2
78.6
77.4
77.4
77.2
76.9
72.4
30.1
28.8
f1 (ppm)

Tetra Boc Amine Alkyne Scaffold [2]

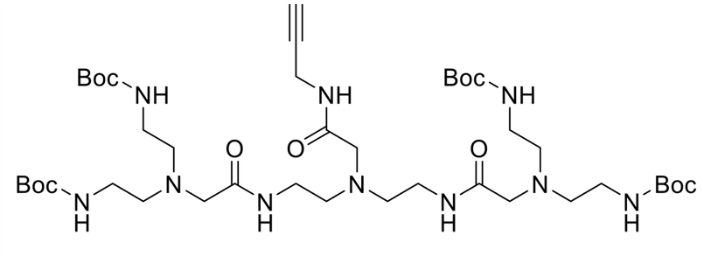

A suspension of tetra Boc amine scaffold (300 mg, 0.38 mmol) and K_2CO_3 (105 mg, 0.76 mmol) in anh. MeCN (5 mL) was cooled to 0 °C. To the stirring suspension was slowly added 2-bromo-*N*-(prop-2-yn-1-yl)acetamide (84 mg, 0.49 mmol) in anh. MeCN (0.5 mL). After addition was complete, the reaction was allowed to warm to 22 °C and stirred under an inert atmosphere for 24 h. After this time, the solvent was removed *in vacuo* and the crude product purified by flash column chromatography (0-10% MeOH/EtOAc) to give tetra Boc amine alkyne scaffold Error! Reference source not found. (193 mg, 0.23 mmol, 59%) as a white solid.

^{1}H NMR (500 MHz, $CDCl_3$) δ 7.79 (br s, 2H), 5.74 (br s, 3H), 4.02 (dd, *J* = 5.6, 2.6 Hz, 2H), 3.32 (q, *J* = 6.0 Hz, 4H), 3.23 (s, 2H), 3.16 – 3.12 (m, 9H), 2.70 (d, *J* = 6.0 Hz, 4H), 2.56 (t, *J* = 5.6 Hz, 8H), 2.22 (d, *J* = 2.6 Hz, 1H), 1.41 (s, 37H).

^{13}C NMR (126 MHz, $CDCl_3$) δ 172.1 (C), 171.5 (C), 156.7 (C), 79.4 (C), 71.5 (CH), 59.4 (CH_2), 55.8 (CH_2), 38.8 (CH_2), 29.8 (CH_2), 28.6 (CH_3).

IR (solid) 3368, 3303, 2976, 2930. 2904, 2824, 1681. 1657, 1523 cm^{-1}.

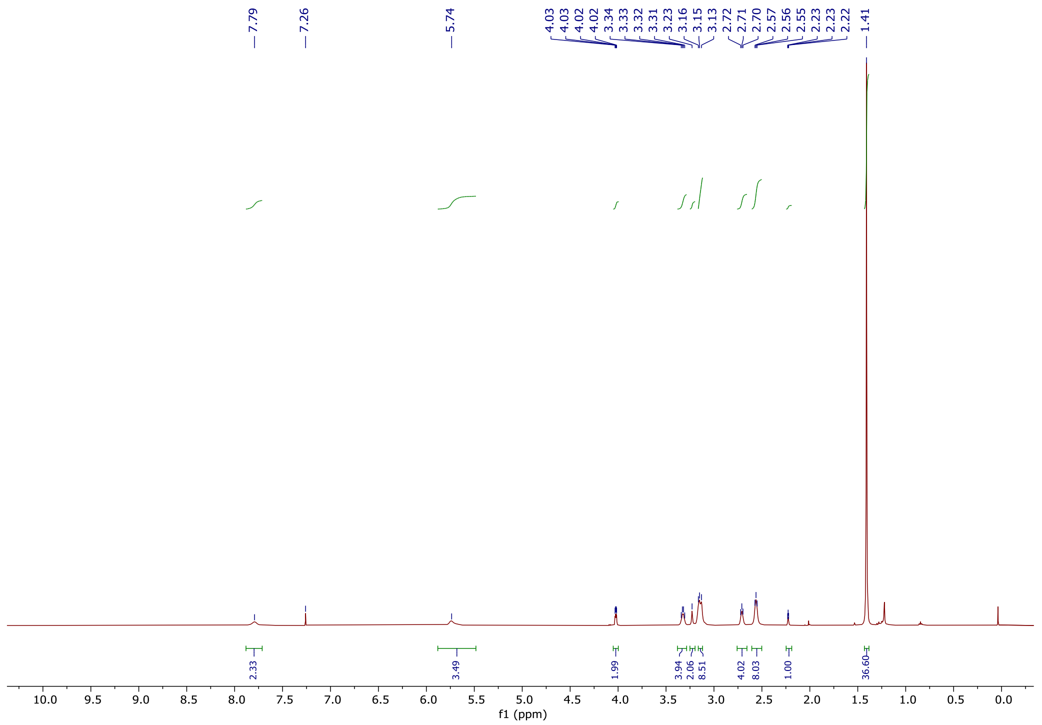
7.79
7.26
5.74
4.03
4.03
4.02
4.02
3.34
3.33
3.32
3.31
3.23
3.16
3.15
3.13
2.72
2.71
2.70
2.57
2.56
2.55
2.23
2.23
2.22
1.41
2.33
3.49
1.99
3.94
2.06
8.51
4.02
8.03
1.00
36.60
f1 (ppm)

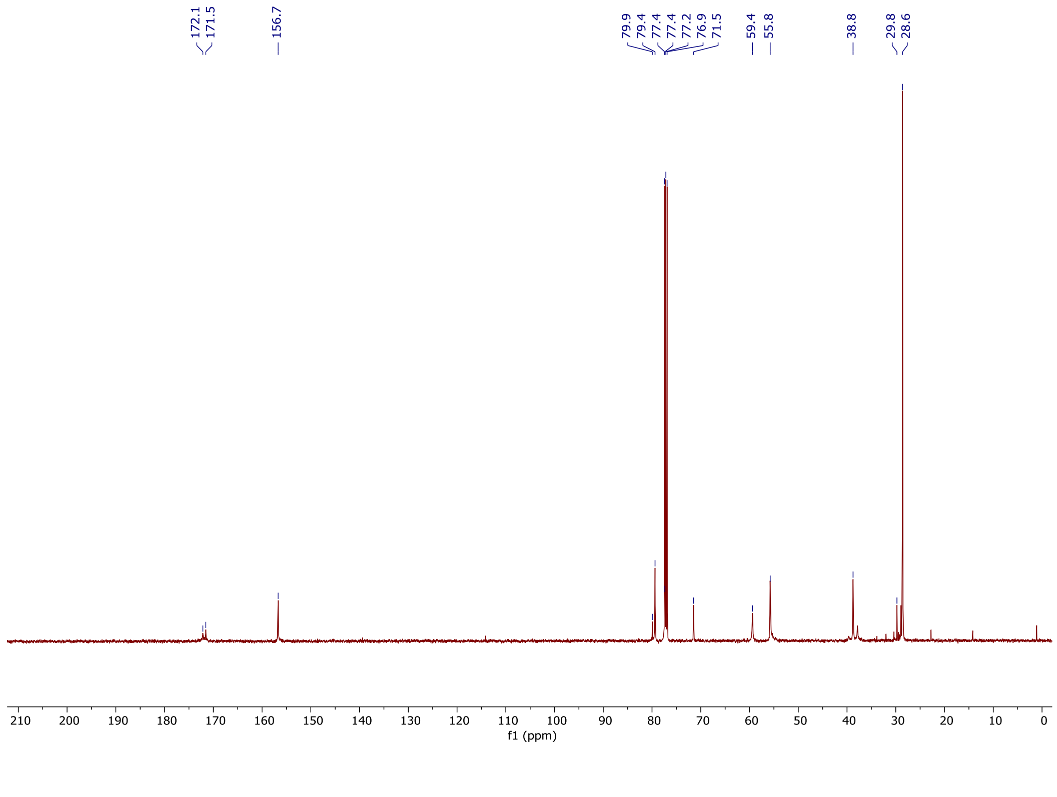
172.1
171.5
156.7
79.9
79.4
77.4
77.4
77.2
76.9
71.5
59.4
55.8
38.8
29.8
28.6
f1 (ppm)

Alkyne TetraPD

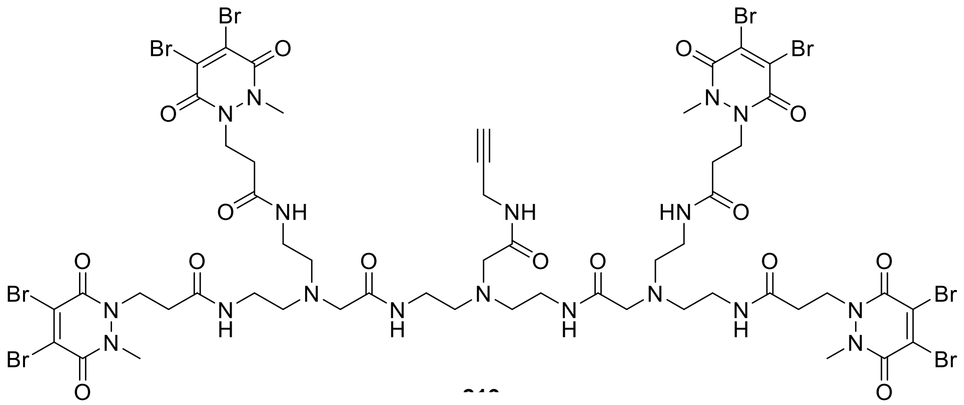

To a solution of tetra Boc amine alkyne scaffold (153 mg, 0.18 mmol) in DCM (1 mL) was added HCl (4 M in dioxane, 2 mL) and the reaction stirred at 22 °C under an inert atmosphere for 16 h. After this time the solvent was removed *in vacuo* and the amine salt re-dissolved in DCM (1 mL, does not dissolve well) and DIPEA (141 µL, 0.79 mmol). This was added dropwise at 0 °C to a solution of MetPAc (281 mg, 0.79 mmol) in DCM (1 mL), pre-activated with EDC.HCl (151 mg, 0.79 mmol) at 0 °C for 30 min. The reaction was allowed to warm to 22 °C and stirred under an inert atmosphere for 24 h. After this time, solvent was removed *in vacuo* and crude product purified *via* flash column chromatography (reverse phase, 0-50% MeCN/water) the alkyne tetraPD (64.3 mg, 0.04 mmol, 20%) as a white solid.

^{1}H NMR (600 MHz, CD_3CN) δ 4.34 (t, *J* = 7.1 Hz, 8H), 3.94 – 3.91 (m, 2H), 3.60 (s, 12H), 3.56 (d, *J* = 1.6 Hz, 2H), 3.22 (t, *J* = 6.1 Hz, 4H), 3.16 (t, *J* = 5.8 Hz, 8H), 3.05 (s, 4H), 2.61 (q, *J* = 6.7 Hz, 4H), 2.56 (t, *J* = 7.1 Hz, 8H), 2.52 (t, *J* = 5.9 Hz, 8H), 2.45 (t, *J* = 2.5 Hz, 1H).*$CHCl_3$ in NMR

^{13}C NMR (151 MHz, CD_3CN) δ 172.4 (C), 170.9 (C), 170.6 (C), 154.0 (C), 153.8 (C), 136.6 (C), 136.2 (C), 79.1 (C), 71.9 (CH), 59.8 (CH_2), 56.3 (CH_2), 55.5 (CH_2), 45.1 (CH_2), 41.3 (CH_2), 38.4 (CH_2), 38.2 (CH_2), 35.7 (CH_3), 34.5 (CH_2), 34.5 (CH_2).

LRMS (ESI) 1829 (5, $[M^{79}Br_8+H]^+$), 1831 (20, $[M^{79}Br_7{}^{81}Br+H]^+$), 1833 (45, $[M^{79}Br_6{}^{81}Br_2+H]^+$), 1835 (100, $[M^{79}Br_5{}^{81}Br_3+H]^+$), 1837 [100, $M^{79}Br_4{}^{81}Br_4+H]^+$, 1839 (80, $[M^{79}Br_3{}^{81}Br_5+H]^+$), 1841 (45, $[M^{79}Br_2{}^{81}Br_6+H]^+$), 1843 (30, $[M^{79}Br_1{}^{81}Br_7+H]^+$), 1845 (15, $[M^{81}Br_8+H]^+$); **HRMS (ESI)** calcd for $C_{53}H_{69}O_{15}N_{18}{}^{79}Br_4{}^{81}Br_4$ $[M^{79}Br_4{}^{81}Br_4+H]^+$ 1836.8569; observed 1826.6342.

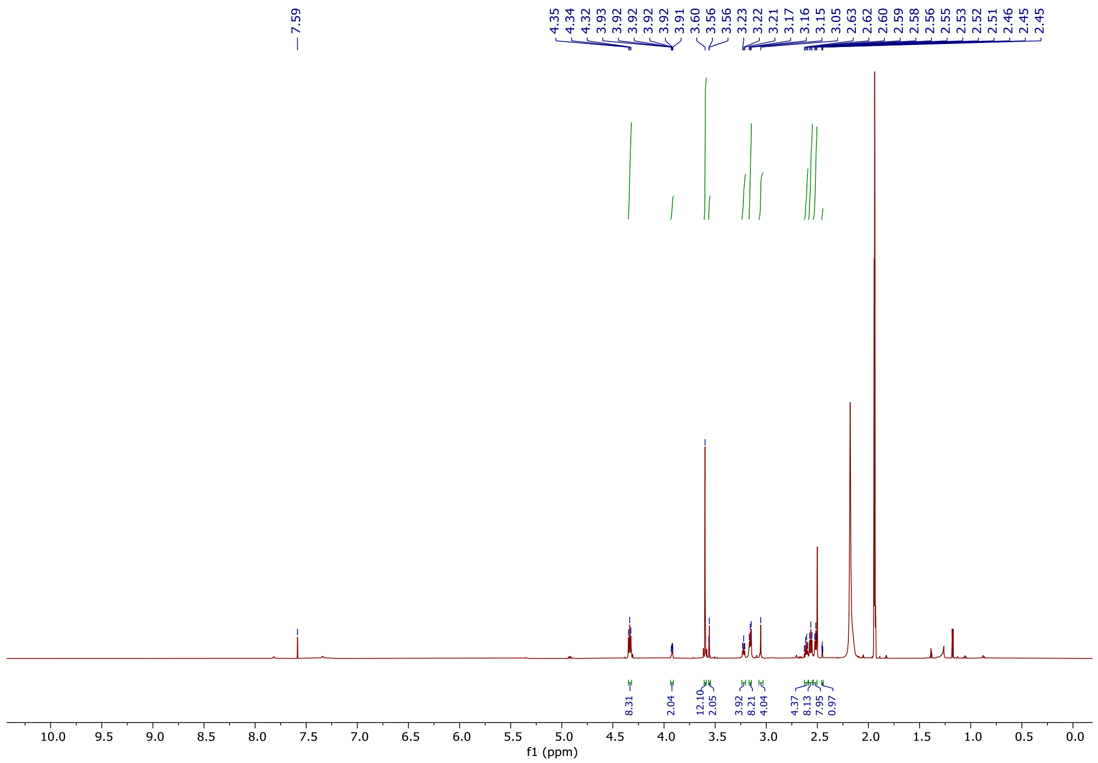
7.59
4.35 4.34 4.32 3.93 3.92 3.92 3.92 3.92 3.91 3.60 3.56 3.56 3.23 3.22 3.21 3.17 3.16 3.15 3.05 2.63 2.62 2.60 2.59 2.58 2.56 2.55 2.53 2.52 2.51 2.46 2.45 2.45
8.31 2.04 12.10 2.05 3.92 8.21 4.04 4.37 8.13 7.95 0.97
10.0 9.5 9.0 8.5 8.0 7.5 7.0 6.5 6.0 5.5 5.0 4.5 4.0 3.5 3.0 2.5 2.0 1.5 1.0 0.5 0.0
f1 (ppm)

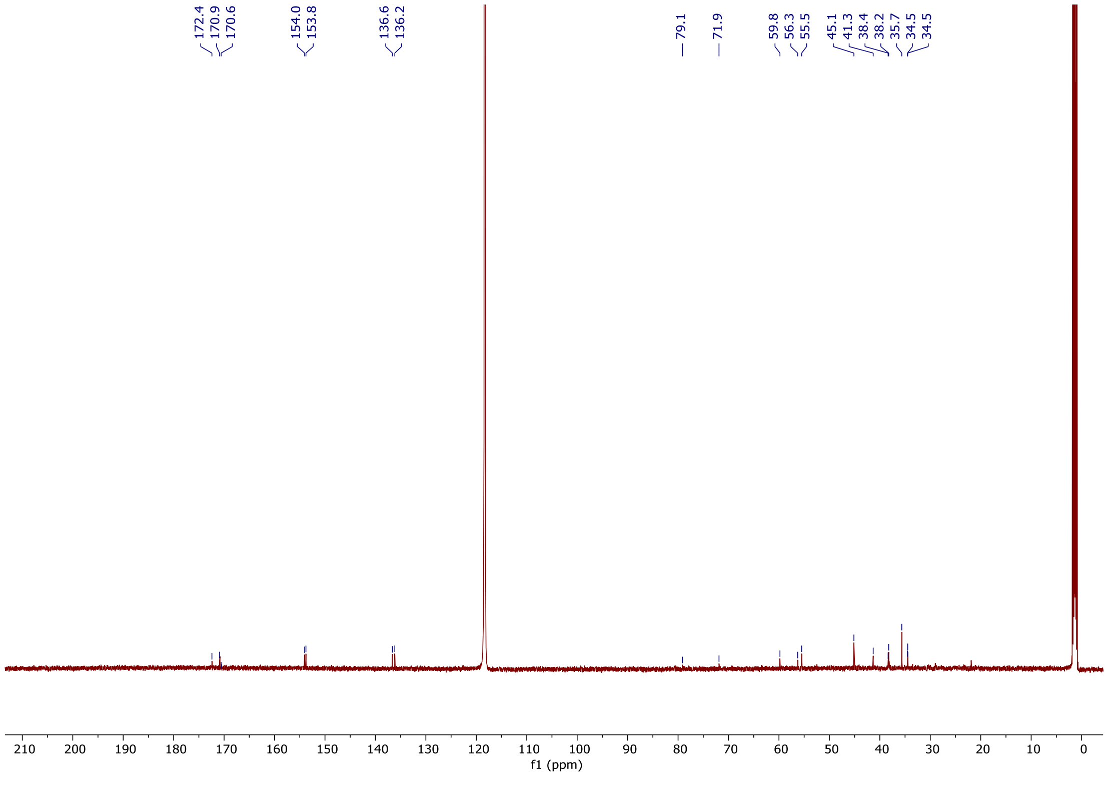
172.4 170.9 170.6
154.0 153.8
136.6 136.2
79.1
71.9
59.8 56.3 55.5
45.1 41.3 38.4 38.2 35.7 34.5 34.5
210 200 190 180 170 160 150 140 130 120 110 100 90 80 70 60 50 40 30 20 10 0
f1 (ppm)

Tetrazine TetraPD 3

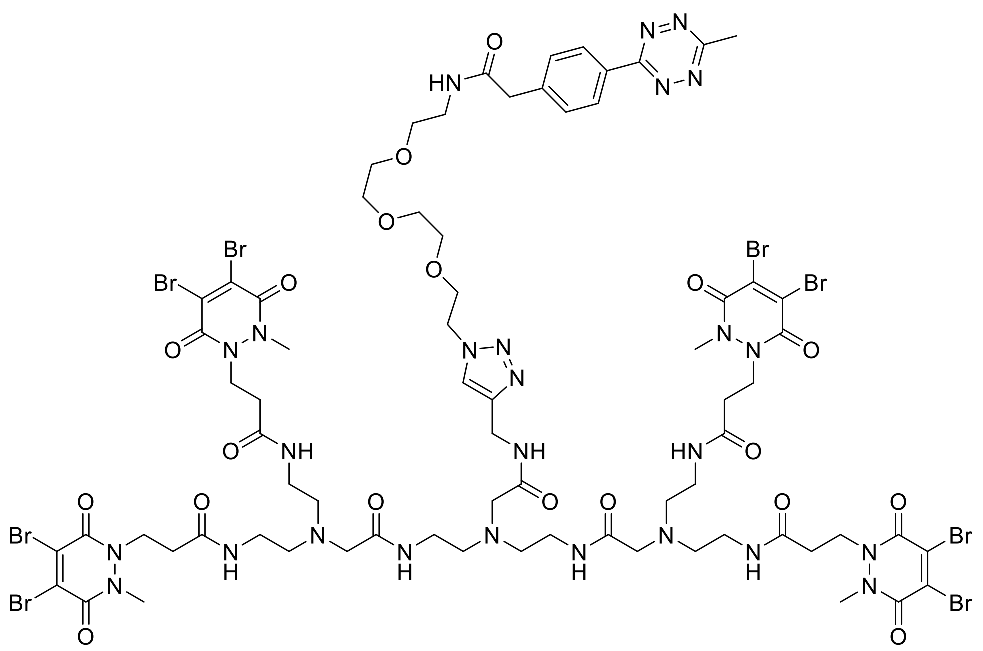

To a solution of alkyne tetraPD (95 mg, 0.05 mmol) in DMF (6 mL) was added CuI (10 mg, 0.05 mmol) and azide tetrazine linker (22 mg, 0.05 mmol) the reaction stirred at 22 °C for 24 h. After this time the solvent was removed *in vacuo* and the crude product purified *via* flash column chromatography (reverse phase, 0-40% MeCN/water) to give Tz tetraPD **3** (11.2 mg, 0.01 mmol, 10%) as a purple solid.

^{1}H NMR ^{1}H NMR (600 MHz, DMSO) δ 8.39 (d, J = 8.3 Hz, 1H), 8.31 – 8.26 (m, 1H), 8.26 – 8.22 (m, 1H), 8.00 (t, J = 5.7 Hz, 4H), 7.91 (d, J = 1.3 Hz, 1H), 7.81 (t, J = 6.0 Hz, 2H), 7.76 (d, J = 8.1 Hz, 1H), 7.53 (d, J = 8.1 Hz, 1H), 7.45 (dd, J = 8.0, 5.6 Hz, 1H), 4.47 (t, J = 5.2 Hz, 2H), 4.32 (d, J = 5.8 Hz, 2H), 4.26 (t, J = 7.2 Hz, 8H), 3.79 (t, J = 5.3 Hz, 2H), 3.65 (s*, 2H), 3.54 (s, 12H), 3.52 – 3.45 (m, 10H), 3.43 – 3.38 (m, 6H), 3.24 – 3.18 (m, 4H), 3.17 – 3.13 (m, 4H), 3.07 (q, J = 6.4 Hz, 8H), 3.02 (s, 3H), 2.99 (s, 2H), 2.56 (d, J = 7.5 Hz, 4H), 2.45 (d, J = 7.2 Hz, 12H).

*Apparent singlet as splitting not resolved

^{13}C NMR (151 MHz, DMSO) δ 174.3 (C), 170.8 (C), 170.6 (C), 169.2 (C), 167.1 (C), 163.2 (C), 152.7 (C), 152.3 (C), 144.8 (C), 135.3 (C), 135.0 (C), 132.1 (CH), 130.1 (C), 130.0 (CH), 129.7 (C), 127.3 (CH), 126.2 (C), 123.1 (CH), 69.7 (CH_2), 69.6 (CH_2), 69.5 (CH_2), 69.1 (CH_2), 68.8 (CH_2), 66.9 (CH_2), 58.1 (CH_2), 54.2 (CH_2), 53.9 (CH_2), 49.3 (CH_2), 42.1 (CH_2), 38.8 (CH_2), 38.8 (CH_2), 36.8 (CH_2), 36.7 (CH_2), 34.7 (CH_3), 34.2 (CH_2), 33.2 (CH_2), 31.3 (CH_2), 28.7 (CH_2), 28.6 (CH_2), 20.9 (CH_3).

IR (solid) 3284, 3081, 2923, 2857, 1628, 1572, 1546 cm^{-1}.

LRMS (ESI) 1130 (2, $[M^{79}Br_8+H]^+/2$), 1131 (10, $[M^{79}Br_7{}^{81}Br+H]^+/2$), 1132 (35, $[M^{79}Br_6{}^{81}Br_2+H]^+/2$), 1133 (70, $[M^{79}Br_5{}^{81}Br_3+H]^+/2$), 1134 (100, $[M^{79}Br_4{}^{81}Br_4+H]^+/2$), 1135 (60, $[M^{79}Br_3{}^{81}Br_5+H]^+/2$), 1136 (50, $[M^{79}Br_2{}^{81}Br_6+H]^+/2$), 1137 (25, $[M^{79}Br_1{}^{81}Br_7+H]^+/2$), 1138 (15, $[M^{81}Br_8+H]^+/2$); **HRMS (ESI)** calcd for $C_{72}H_{96}Br_8N_{26}O_{19}/2$ $[M^{79}Br_4{}^{81}Br_4]^+/2$ 1134.0360; observed 1134.0324.

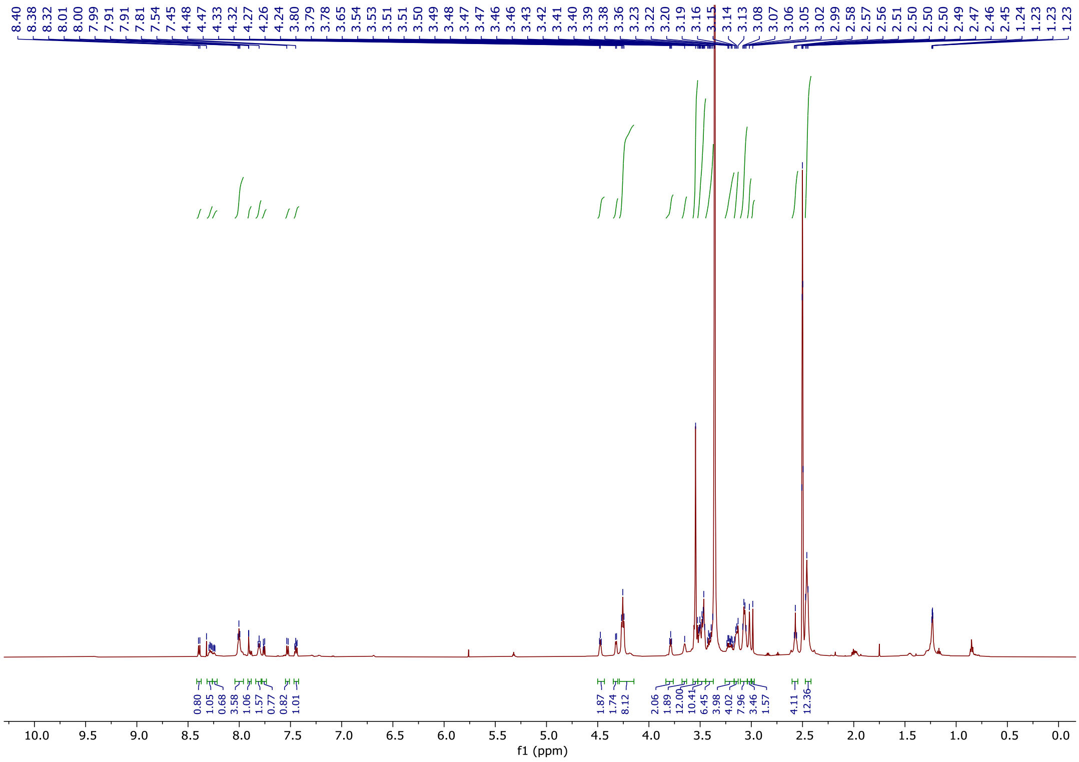

COSY

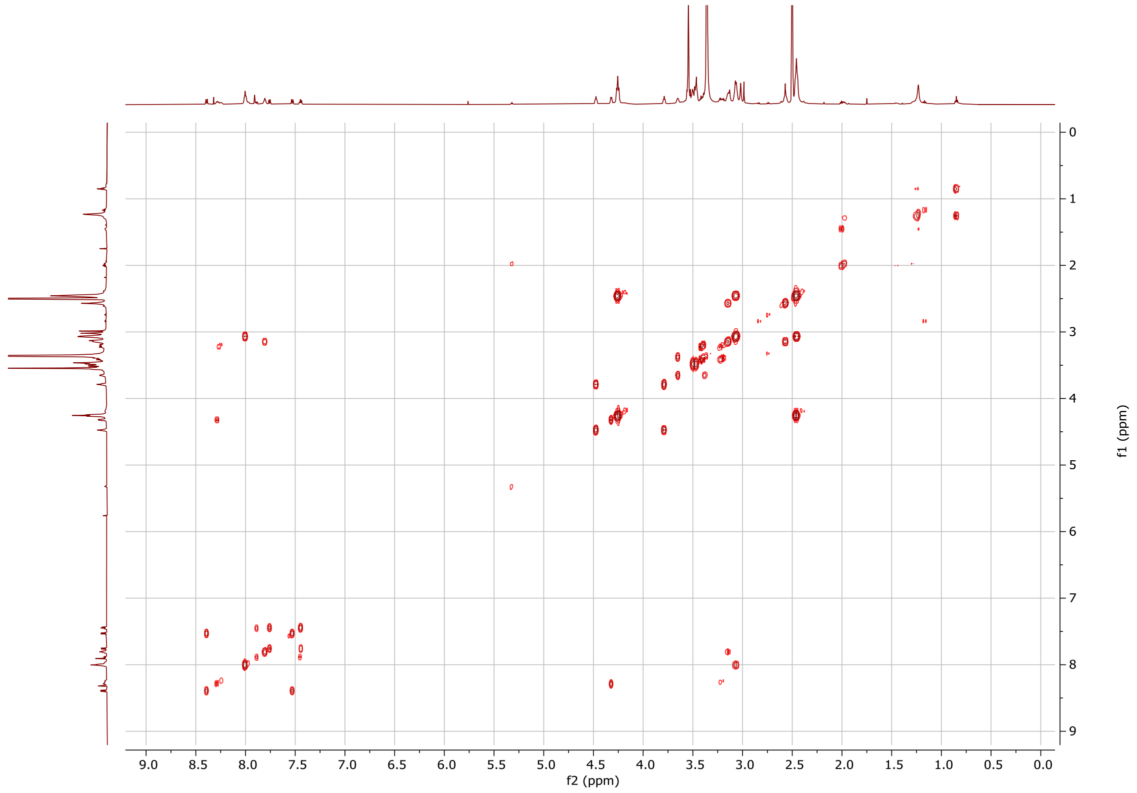

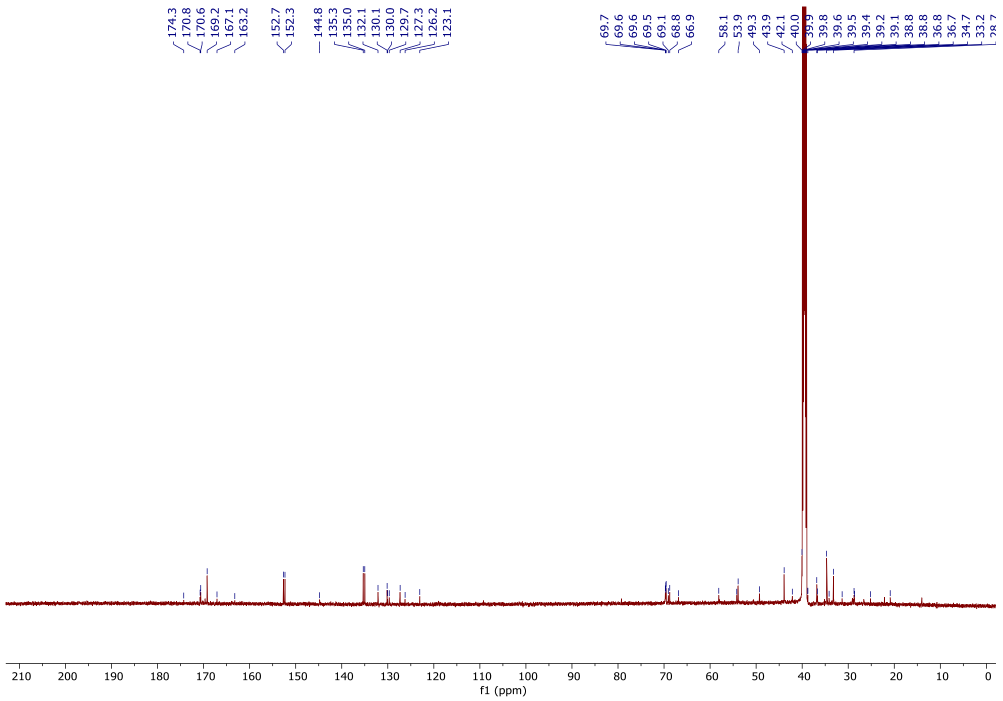

HSQC

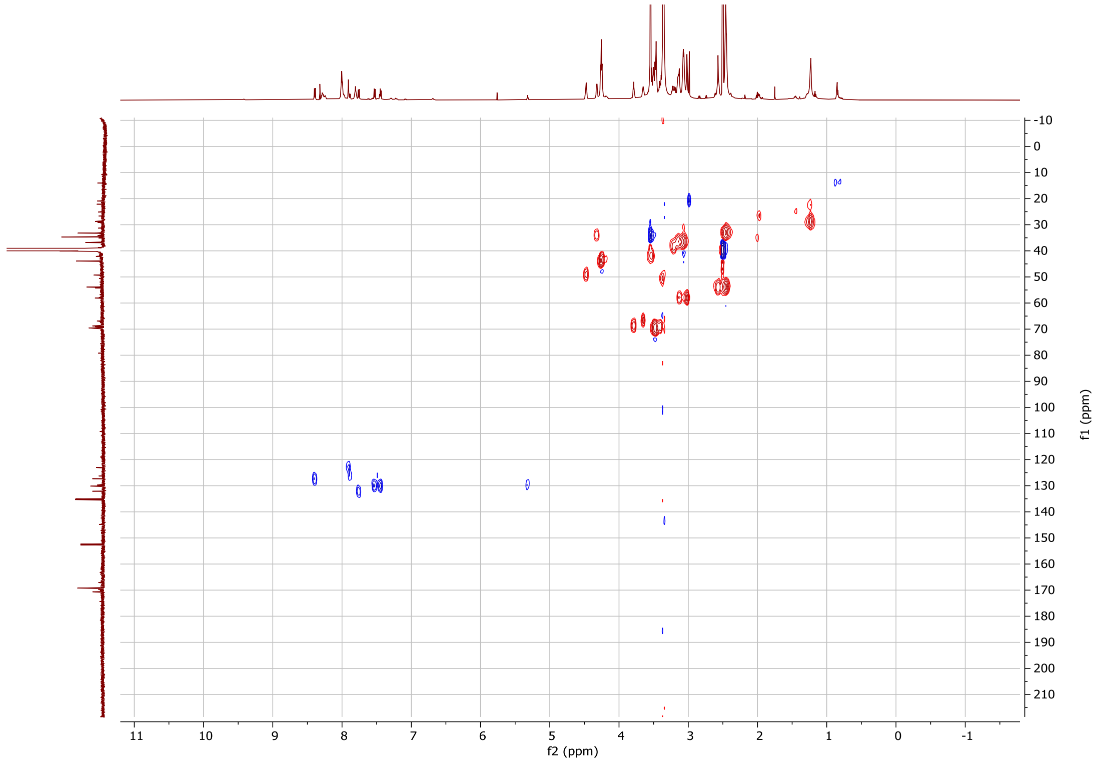

Antibody–DNA conjugation

Method 1 – BCN PD-mediated conjugation

BCN PD (0.3 µL, 20 mM in DMSO) was combined with 5'-MeTz-PEG$_5$-ACCACCA-3' (1.2 µL, 10 mM in TE buffer: 10 mM Tris, 1 mM EDTA, pH 8.0; 2-fold molar excess relative to BCN PD) in nuclease-free borate buffered saline (25 mM borate, 25 mM NaCl, 2 mM EDTA, pH 8.0; 15 µL total volume). The mixture was incubated at 37 °C with constant agitation (300 rpm) for 20 h to generate the clicked oligonucleotide–PD.

The next day, anti-GFP antibody (15 µL, 40 µM) in borate buffered saline (25 mM borate, 25 mM NaCl, 2 mM EDTA, pH 8.0) was reduced by addition of TCEP·HCl (0.3 µL, 20 mM in deionized water, 10-fold molar excess) and incubated at 37 °C with constant agitation (300 rpm) for 1.5 h. Excess TCEP was removed using a 7 kDa MWCO Zeba Spin Desalting Column (Thermo Fisher Scientific).

The resulting pre-clicked oligonucleotide–PD conjugate **1** (15 µL, 200 µM) was added to reduced anti-GFP antibody (15 µL, 40 µM; final antibody concentration 20 µM) and the reaction incubated at 37 °C with constant agitation (300 rpm) for 20 h. Unreacted material was removed by centrifugal filtration, yielding conjugate **2** (expected mass = 157,782 Da; observed mass = 157,773 Da). The final concentration and oligonucleotide-to-antibody ratio (OAR) were determined by LC–MS and UV–Vis spectroscopy.

Method 2 – Tz-tetra-PD mediated re-bridging conjugation

Anti-GFP antibody (100 µL, 20 µM) in borate buffer was reduced by addition of TCEP·HCl (1 µL, 20 mM in deionized water, 10-fold molar excess) and incubated at 37 °C with constant agitation (300 rpm) for 1.5 h. Excess TCEP was removed using a 7 kDa MWCO Zeba Spin Desalting Column (Thermo Fisher Scientific), and the reduced antibody was reacted with Tz-tetraPD **3** (0.5 µL, 20 mM in DMSO, 5-fold molar excess) at 37 °C with constant agitation (300 rpm) for 3 h. Unreacted small molecule was removed by centrifugal filtration, yielding conjugate **4** (expected mass = 146,708 Da; observed mass = 146,702 Da). The concentration and protein-to-dye ratio (PDAR) were determined by LC–MS and UV–Vis spectroscopy.

The resulting anti-GFP–PD conjugate **4** (14 µL, 11.5 µM) in borate buffered saline was subsequently reacted with 5′-TCO-PEG$_4$-ACCACCACCACCACCA-3′; custom-synthesized by Eurofins (0.4 µL, 2 mM in TE buffer). The reaction mixture was incubated at 37 °C with constant agitation (300 rpm) for 16 h. Following incubation, unreacted material was removed using a 10 kDa centrifugal filter, affording conjugate **5** (expected mass = 151,954 Da; observed mass = 151,945 Da). The final concentration and oligonucleotide-to-antibody ratio (OAR) were determined by LC–MS and UV–Vis spectroscopy.

Method 3 – Maleimide–PEG_2–succinimidyl ester conjugation[3]

Anti-GFP antibody (37 µL, 18 µM) in PBS buffer was reacted with maleimide–PEG2–succinimidyl ester (0.3 µL, 23.5 mM in deionized water, 10-fold molar excess) at 4 °C with constant agitation (300 rpm) for 1.5h in the dark. Excess unreacted cross-linkers were removed using a 7 K MWCO Zeba spin desalting column (Thermo Fisher Scientific).

In parallel, DNA-PAINT docking strand 5'-Thiol- AAACCACCACCACCACCA-3' (13 µL, 1 mM in PBS buffer) was reduced in DTT buffer (250 mM DTT, 1.5 mM EDTA and 0.5× PBS, pH 7.2). The mixture was incubated at room temperature with constant agitation (300 rpm) for 2h. Excess DTT was eliminated using Microspin Illustra G-25 column (GE Healthcare).

The reduced DNA strand was subsequently added to the activated antibody (10-fold molar excess of DNA to antibody) and incubated overnight at 4 °C with constant agitation (300 rpm) in the dark. Following incubation, residual free DNA was removed using a 100 kDa centrifugal filter. The final concentration and oligonucleotide-to-antibody ratio (OAR) were determined by LC–MS and UV–Vis spectroscopy.

Liquid Chromatography–Mass Spectrometry (LCMS)

Molecular masses of all protein conjugates were determined using an Agilent 6530 QTOF LCMS system (Agilent Technologies, UK). Samples (2 µL, 0.2 mg/mL in LCMS grade water) were injected onto an Agilent 1290 Infinity II UHPLC equipped with an Agilent PLRP-S column (1000 Å, 8 µm, 50 × 2.1 mm). Chromatographic separation was achieved at 60 °C using mobile phase A (water, 0.1% formic acid) and mobile phase B (acetonitrile, 0.1% formic acid) at a flow rate of 0.8 mL/min. A linear gradient was applied from 20% to 60% B over 6.5 min, held for 1 min, then returned to 20% B and re-equilibrated for 1 min.

The mass spectrometer was operated in positive polarity mode with an electrospray ionization (ESI) source under the following conditions: capillary voltage, 4000 V; gas temperature, 350 °C; drying gas flow rate, 10 L/min; nebulizer pressure, 35 psig; fragmentor voltage, 175 V; skimmer voltage, 65 V. Data were acquired in profile mode at 1 spectrum/s over an m/z range of 100–7000. Raw spectra were deconvoluted to zero-charge mass spectra using the maximum entropy algorithm in Agilent MassHunter software (version B.07.00).

For deglycosylation, antibody samples (40 µL, 5 µM in water) were incubated with PNGase F (0.67 µL; New England BioLabs) at 22 °C for 16 h prior to analysis. Representative chromatograms and deconvoluted spectra (total ion chromatogram, raw mass trace, and deconvoluted spectra) are provided in the Supporting Information.

UV–Vis Spectroscopy

UV–Vis absorption spectra were recorded on a NanoDrop™ One microvolume spectrophotometer (Thermo Fisher Scientific) at room temperature. Sample buffer was used as the reference for baseline correction. Antibody concentrations and conjugation ratios were determined using Beer–Lambert law–based quantification at λ_{max} values corresponding to each chromophore. Extinction coefficients for antibodies (ε_{280}) and conjugated payloads ($\varepsilon_{\lambda max}$) were obtained from manufacturer datasheets (i.e., PD scaffold, ε_{335} = 9,100 M^{-1} cm^{-1}; 5'-MeTz-PEG_5-ACCACCA-3', ε_{260} = 76,200 M^{-1} cm^{-1}; 5′-TCO-PEG_4-ACCACCACCACCA-3′, ε_{260} = 167,400 M^{-1} cm^{-1}). Where no literature correction factor was available, an empirical value was determined using NanoDrop™ measurements. Where applicable, correction factors were applied to account for overlapping absorbance at 280 nm (i.e., PD scaffold, correction factor at A_{280} = 0.25). Concentration (c) and PD to antibody ratio (PDAR) of conjugates was calculated by UV-Vis using the following equations as previously described[4,5]:

$$A_{280} = c(\varepsilon_{280(Ab)} + 0.25 \cdot n \cdot \varepsilon_{335(PD)})$$
$$A_{260} = c(\varepsilon_{260(Ab)} + n \cdot \varepsilon_{260(oligo)})$$
$$PDAR = \frac{\varepsilon_{280(Ab)} \cdot A_{335}}{A_{280} \cdot \varepsilon_{335(PD)} - 0.25 \cdot A_{335} \cdot \varepsilon_{335(PD)}}$$

SDS-PAGE

Non-reducing Tris-glycine SDS-PAGE was conducted using a precast gel (15% acrylamide, 4% stacking; BioRad™) according to standard protocols. A broad-range molecular weight marker (10–250 kDa, PageRuler™ Plus Pre-stained Protein Ladder, Thermo Scientific™) was included for mass estimation. Antibody-DNA conjugates (10 µM, 5 µL) were mixed 1:1 with 5× loading buffer (8 mL of glyerol, 4 mL of deionized water, 1.6 mL of 10% (w/v) SDS, 1 mL of 0.5 M Tris HCl pH 6.8 buffer, 0.2 mL of 0.5% (w/v) bromophenol blue solution) and heated at 85 °C for 5 min. Following incubation, 6 µL of each sample was loaded per well. Electrophoresis was carried out at 170 V, 400 mA for 50 min in 1 × SDS running buffer. Gels were stained with Coomassie Brilliant Blue and destained in deionized water under microwave irradiation.

Cell Culture and Nup96 Labeling

U2OS CRISPR mEGFP–Nup96 cells were maintained in McCoy's 5A medium (Thermo Fisher Scientific, 16600082) supplemented with 10% fetal bovine serum (FBS) and 1% penicillin–streptomycin. Cultures were kept at a density of approximately 1×10^5 cells/ml and passaged using Accutase (PromoCell, C-41310).

For imaging experiments, 3×10^4 cells were seeded into 8-well glass-bottom Ibidi slides (IB-80807) 24 h before fixation. After removing the growth medium, cells were fixed in 4%

paraformaldehyde (PFA) for 30 min at room temperature. Permeabilization was carried out with 0.1% Triton X-100 for 5 min, followed by three 3-min washes with 60 mM glycine in PBS to quench autofluorescence. Samples were blocked with 5% bovine serum albumin (BSA) for 1 h in the dark.

For specific labeling of Nup96, cells were incubated with 20 nM of either the three different DNA-conjugated anti-GFP nanobody, prepared as described previously, or DNA coupled anti-GFP nanobody (Massive-sdAB-FAST 2-Plex, Massive Photonics GmbH) in 5% BSA for 1 h at room temperature. Excess antibody or nanobody was removed by three 3-min washes with PBS. Imaging was performed using 0.3 nM DNA imager strand (either R2: TGG TGG T-Cy3B, Eurofins or F3-Cy3B, for Massive-sdAB-FAST 2-Plex) in C+ buffer with or without 1× Trolox, 1× PCA, and 1× PCD supplement.

Total Internal Reflection Fluorescence (TIRF) Microscopy

TIRF imaging was performed on a custom-built setup based on a Nikon Eclipse Ti2 microscope equipped with a 100× oil-immersion TIRF objective (Apo TIRF, NA 1.49) and Nikon's Perfect Focus System. Excitation for DNA-PAINT was achieved using a 560 nm laser (1 W, MPB Communications) configured for flat-top illumination. A 488 nm laser (OBIS 488 nm LX, 150 mW) that shares the same beam path as the 561 nm laser was used to excite the mGFP-Nup96 of the used cells. The laser beam was expanded using a custom telescope combined with a variable beam expander, then passed through a beam shaper (piShaper 6_6_VIS, AdlOptica, Berlin, Germany) to convert the Gaussian profile into a collimated, uniform intensity distribution. Circular polarization was generated via a linear polarizer followed by a quarter-wave plate. The beam was focused into the back focal plane of the objective lens using an appropriate focusing lens, transmitted through an excitation filter (FF01-390/482/563/640-25, Semrock), and directed into the objective via a multiband dichroic mirror (Di03-R405/488/561/635, Semrock).

Fluorescence emission was collected through the same objective, spectrally filtered with an emission filter (FF01-446/523/600/677-25, Semrock), and detected on a sCMOS camera (ORCA-Fusion BT, Hamamatsu). No additional magnification was applied, resulting in an effective pixel size of 130 nm in the sample plane after 2×2 binning.

During image acquisition, the flattest cells were first identified in brightfield. The 488 nm laser was then used to locate the focal plane of the NUP96 rings (GFP signal) and to verify cell flatness by ensuring a complete nucleus was visible within a single axial position. Nup96-labeled U2OS cells were imaged over 20,000 frames with an integration time of 100 ms per frame and an excitation intensity of 160 W cm^{-2}.

DNA-PAINT analysis

Raw fluorescence movies were processed for super-resolution reconstruction using the Picasso software package (latest version accessible at https://github.com/jungmannlab/picasso)[3]. Drift correction was performed via redundant cross-correlation.

Super-resolution images were generated using Picasso's Render module. Individual localizations were depicted as Gaussian spots, with widths corresponding to their localization precision. For each pixel, the intensities of overlapping Gaussian spots were summed, and the resulting image was visualized using an appropriate colormap.

Localization precision and resolution

Localization precision (σ_{SMLM}) was estimated using the Cramér–Rao lower bound (CRLB) derived from single-molecule fitting. The reported precision corresponds to the median of $\sigma_x$and σ_y, taken as the mode values of their respective distributions. Additionally, localization precision was independently validated using nearest-neighbor analysis (NeNA)[6].

Table S1. Experimental conditions and localization statistics.

Dataset	Buffer	Labeling	NeNa (nm)	Median σ_x (nm)	Median σ_y (nm)	# Localizations per NPC per minute
Fig. 2	Trolox	Nanobody	3.2	3.0	2.8	5 ± 1
Fig. 2	Trolox	OAR4	3.7	2.7	2.6	11 ± 2
Fig. 2	Trolox	OAR1	3.6	3.0	3.0	14 ± 3
Fig. S8	C+	Nanobody	2.8	2.5	2.4	7 ± 2
Fig. 3.b.	C+	OAR4	5.7	5.5	5.1	19 ± 6
Fig. 3.c.	C+	OAR1	4.1	3.6	3.5	18 ± 4
Fig. 3.d.	C+	Lysine-Conjugation	4.8	6.4	6.3	27 ± 4

Quantitative-PAINT analysis

Nup96 DNA-PAINT datasets were analyzed using quantitative PAINT (qPAINT) with a custom Python pipeline to infer the number of labeled proteins per localization cluster. The nuclear pore complex (NPC) exhibits eightfold rotational symmetry, with Nup96 arranged as stable homodimeric pairs at each of the eight symmetry-related positions. Thus, each symmetry position is expected to contain either a single labeled protein (monomer) or a fully labeled dimer. To determine the stoichiometry at each position, the analysis was performed in two sequential steps: spatial clustering followed by temporal trace analysis.

First, localization datasets were clustered using the SMLM clustering algorithm implemented in Picasso (Render module, Postprocess). Clusters were defined using a 15 nm radius centered on the expected Nup96 positions. The 15 nm clustering radius was selected to be sufficiently large to capture all localizations originating from a fully labeled Nup96 dimer at a single symmetry position, while remaining small enough to prevent merging of signals from adjacent positions. Owing to incomplete labeling efficiency, clusters therefore correspond to either singly labeled (monomeric) or doubly labeled (dimeric) Nup96 positions. Positions without detectable localizations or clusters containing less than 10 localizations were excluded from further analysis. Distributions of localizations per NPC per minute and clusters per NPC for the four labeling strategies (Nanobody, OAR4, OAR1, and lysine-based conjugation) are shown in Figure S10a–b. Across all conditions, approximately 5–6 detectable clusters per NPC were observed on average.

For each spatial cluster, localizations were grouped, and their frame numbers used to reconstruct the sequence of dark times, defined as consecutive frames without detected binding events. Dark times for a given cluster were pooled and their mean value calculated. Because dark times follow an exponential distribution, the sample mean is the maximum-likelihood estimator of the characteristic decay constant τ, which represents the dark time for any given cluster of single molecule localizations. The qPAINT index of a cluster was defined as: q = 1/τ. Under the architectural model described above, each cluster arises from either one labelled protein (monomer) or two labelled proteins (dimer). Since the qPAINT index is proportional to the binding frequency, clusters with two binding sites are expected to exhibit approximately twice the q value of single-binding-site clusters. In this ideal case, the q distribution can therefore be modeled as a two-component Gaussian mixture:

$$f(q) = A_1 e^{-\frac{(q-\mu_1)^2}{2\sigma_1^2}} + A_2 e^{-\frac{(q-\mu_2)^2}{2\sigma_2^2}}$$

where μ_1 and μ_2 (~ $2\mu_1$) corresponds to the qPAINT index of a single and doble binding site, respectively. A_1 and A_2 denote the relative weights of each population.

Experimental q distributions, however, deviated from this idealized two-component model, particularly for the random lysine-based conjugation strategy, reflecting kinetic heterogeneity and potential higher apparent binding-site numbers. Therefore, for each labeling condition, qPAINT index histograms were generated from all detected clusters and fitted using a penalized Gaussian mixture model with K components. The probability density function was defined as:

$$f(q) = \sum_{k=1}^{K} A_k \frac{1}{\sqrt{2\pi\sigma_k^2}} \exp\left(-\frac{\left(q - (\mu_0 k + \delta_k)\right)^2}{2\sigma_k^2}\right)$$

where A_k are the mixture weights ($\sum A_k = 1$), σ_k are the standard deviations of each component. To incorporate the expected proportionality between binding-site number and qPAINT index, the component means were parameterized as:

$$\mu_k = \mu_0 k + \delta_k$$

allowing small deviations δ_k from exact integer multiples of a base value μ_0. This parameterization constrains the model around the physically expected linear scaling of q with binding-site number while allowing flexibility to account for experimental variability. Parameters were estimated by minimizing the penalized negative log-likelihood.

For K = 3, the model contained nine free parameters: one base mean μ_0, three deviations δ_k , three standard deviations σ_k , and two independent mixture weights (with the third defined by normalization).

Nanobody, OAR4, and OAR1 datasets were adequately described with K = 3 components, whereas the random lysine-based conjugation dataset required K = 5 components to capture the broader distribution. Fitted parameters (μ_k, σ_k, A_k) are reported in Table S2.

For Nanobody, OAR4, and OAR1, the third component (k=3) contributed <15% of total weight ($A_3 < 0.15$), indicating dominance of two principal populations consistent with one- and two-binding-site clusters. In contrast, the lysine-based conjugation condition exhibited broader, heterogeneous distributions, and individual components could not be directly mapped to discrete oligomeric states.

For each labeling condition, the effective single-binding-site calibration value, $\overline{\mu_1}$, was defined as the mean of the fitted component means:

$$\overline{\mu_1} = \frac{\sum \mu_k}{\sum k}$$

This empirical calibration accounts for deviations from ideal scaling. For each cluster, the normalized ratio $\frac{q}{\overline{\mu_1}}$ was used to assign binding-site numbers. A cluster was classified as containing n binding sites when: $n - 0.5 \leq \frac{q}{\overline{\mu_1}} < n + 0.5$ This procedure was applied independently to all clusters within each NPC. Figure S10c–d shows the distribution of binding sites per NPC and the average number of localizations per binding site. Clusters assigned higher binding-site numbers exhibited proportionally increased localization counts, supporting the internal consistency of the calibration.

For Nanobody, OAR4, and OAR1, one binding site was interpreted as a monomeric labeled protein and two binding sites as a dimeric labeling event. Such direct mapping was not possible for the case of random lysine-based conjugation condition due to more complex binding kinetics. Finally, Figure S10e presents the distribution of the number of labeled proteins per NPC. Assuming a maximum of 16 Nup96 proteins per NPC, labeling efficiency (Q-labeling) was calculated as the mean number of labeled proteins per NPC divided by 16 (see Table S3).

Table S2. Fitted parameters from penalized Gaussian mixture modeling of qPAINT index distributions for each labeling condition. Reported parameters include the fitted peak positions (μ_k, 10^{-2} s^{-1}) standard deviations (σ_k, 10^{-2} s^{-1}), and mixture weights (A_k).

	Nanobody			OAR4			OAR1			Lysine-Conjugation		
k	μ_k	σ_k	A_k	μ_k	σ_k	A_k	μ_k	σ_k	A_k	μ_k	σ_k	A_k
**1**	0.24	0.08	0.45	0.65	0.21	0.38	0.62	0.25	0.45	0.65	0.23	0.27
**2**	0.45	0.15	0.50	1.24	0.38	0.47	1.21	0.42	0.52	1.37	0.26	0.13
**3**	1.05	0.85	0.05	1.98	0.72	0.15	1.97	1.05	0.03	2.06	0.51	0.39
**4**										2.83	0.02	0.02
**5**										3.46	0.87	0.19
$\overline{\mu_1}$	**0.29**			**0.64**			**0.63**			**0.68**		

Table S3. Average number of proteins per nuclear pore complex (NPC) (mean ± standard deviation) and corresponding labeling efficiency for the Nanobody, OAR4, and OAR1 conditions. Labeling efficiency was calculated assuming a maximum of 16 Nup96 molecules per NPC.

Labeling	# Proteins per NPC	Labeling Efficiency
Nanobody	6 ± 2	0.4 ± 1
OAR4	7 ± 2	0.5 ± 0.1
OAR1	7 ± 2	0.5 ± 0.1

Supporting Figures

Native anti-GFP antibody

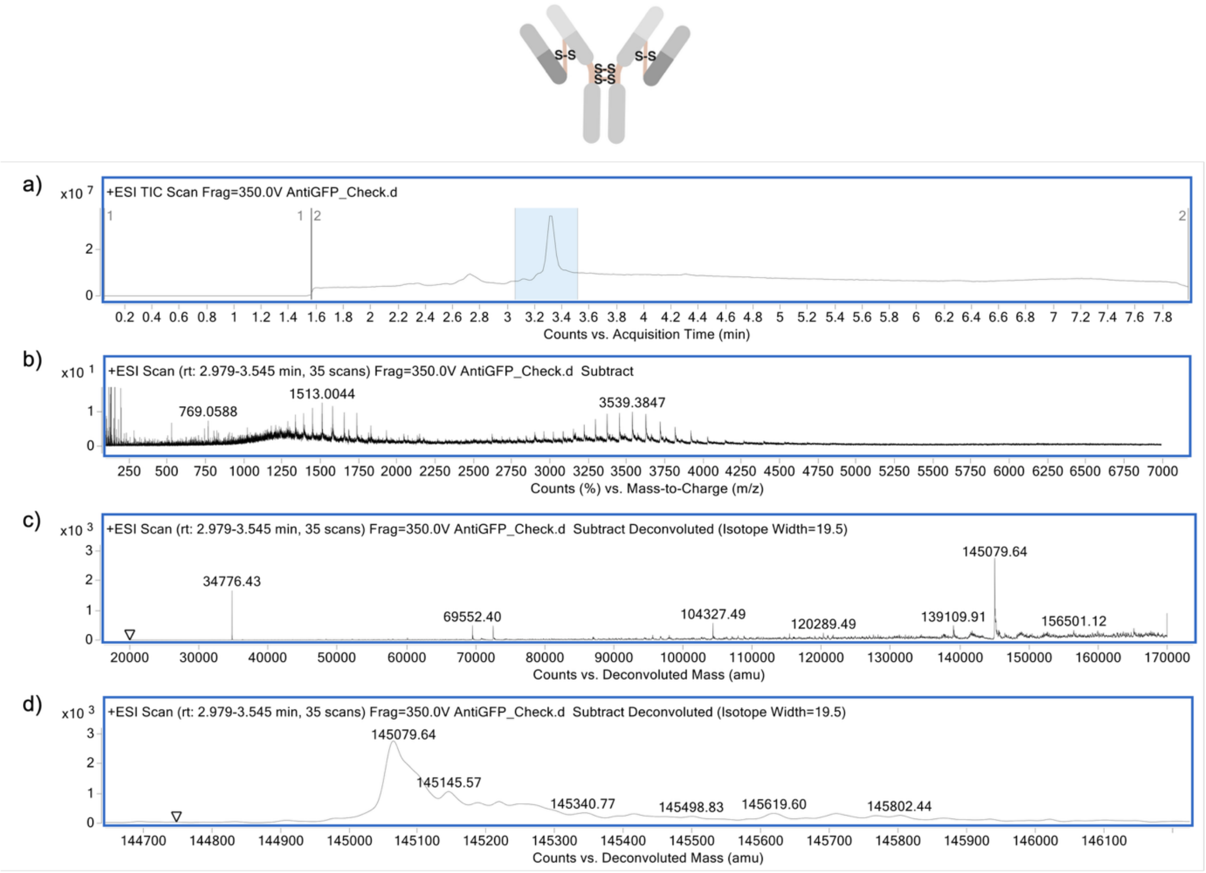

Figure S1. LC-MS spectra for native anti-GFP antibody comprising of the (a) total ion chromatogram, (b) non-deconvoluted ion series, (c) deconvoluted ion series mass spectrum, (d) deconvoluted mass spectrum region of interest; observed mass of 145,080 Da corresponds to native anti-GFP (deglycosylated) antibody.

Anti-GFP-DNA conjugate 2

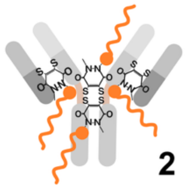

2

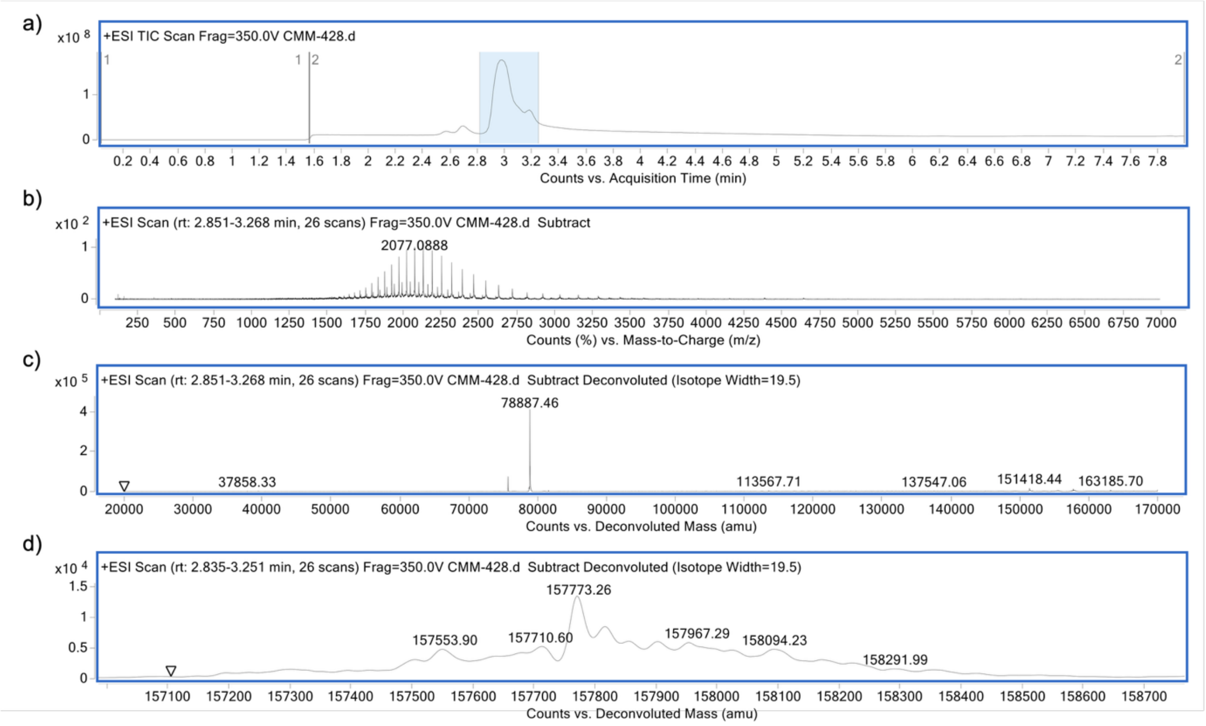

Figure S2. LC-MS spectra for anti-GFP-DNA conjugate **2** observing the addition of expected linker mass (3,175 Da x 4) to the native anti-GFP (145,080 Da), following protein modification with BCN-PD-oligo and purification. (a) Total ion chromatogram, (b) non-deconvoluted ion series, (c) deconvoluted ion series mass spectrum, (d) deconvoluted mass spectrum region of interest; observed mass of 157,773 Da corresponds to 4 BCN-PD-oligo linker additions to anti-GFP antibody (calculated mass = 157,782 Da). Half antibody species is also present; this does not affect OAR. Calculated mass of conjugated half antibody species = 78,891 Da, observed mass = 78,887 Da.

Anti-GFP antibody re-bridged with Tz tetra-PD conjugate 4

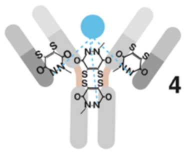

4

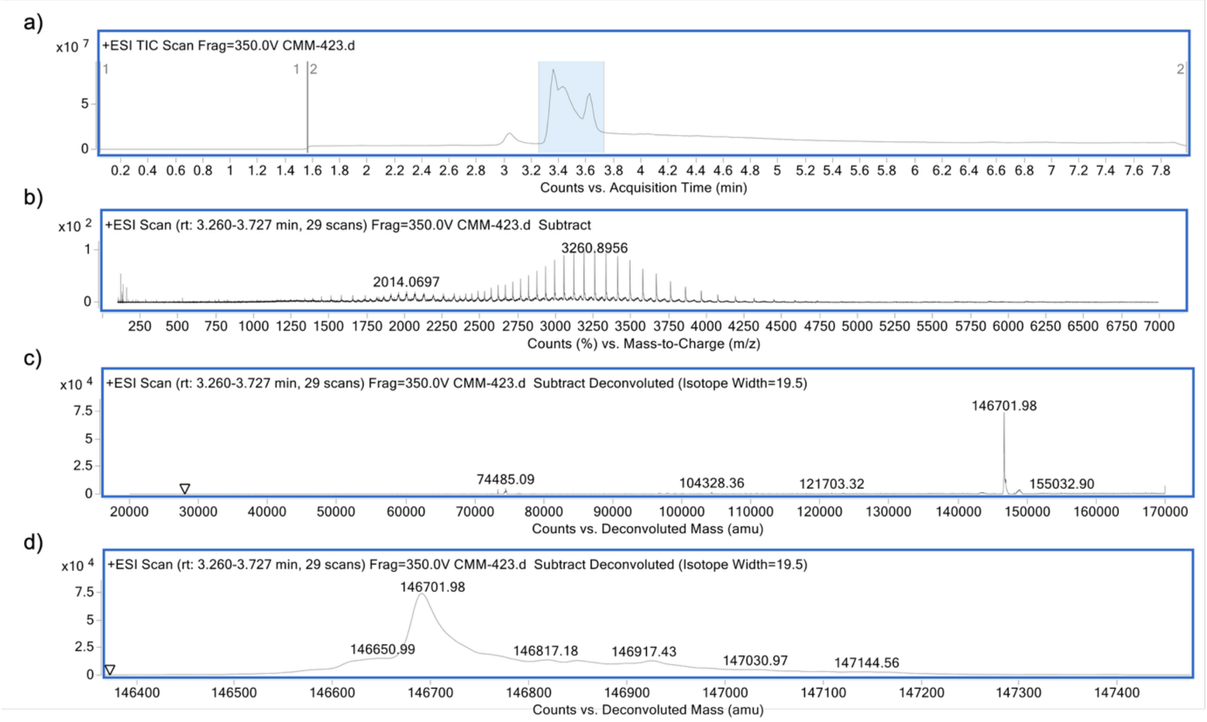

Figure S3. LC-MS spectra for anti-GFP antibody re-bridged with Tz tetra-PD conjugate **4**, comprising of the (a) total ion chromatogram, (b) non-deconvoluted ion series, (c) deconvoluted ion series mass spectrum, (d) deconvoluted mass spectrum region of interest; observed mass of 146,702 Da corresponds to anti-GFP antibody re-bridged with Tz tetra-PD (calculated mass = 146,709 Da).

Anti-GFP–PD–oligo conjugate 5

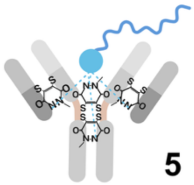

5

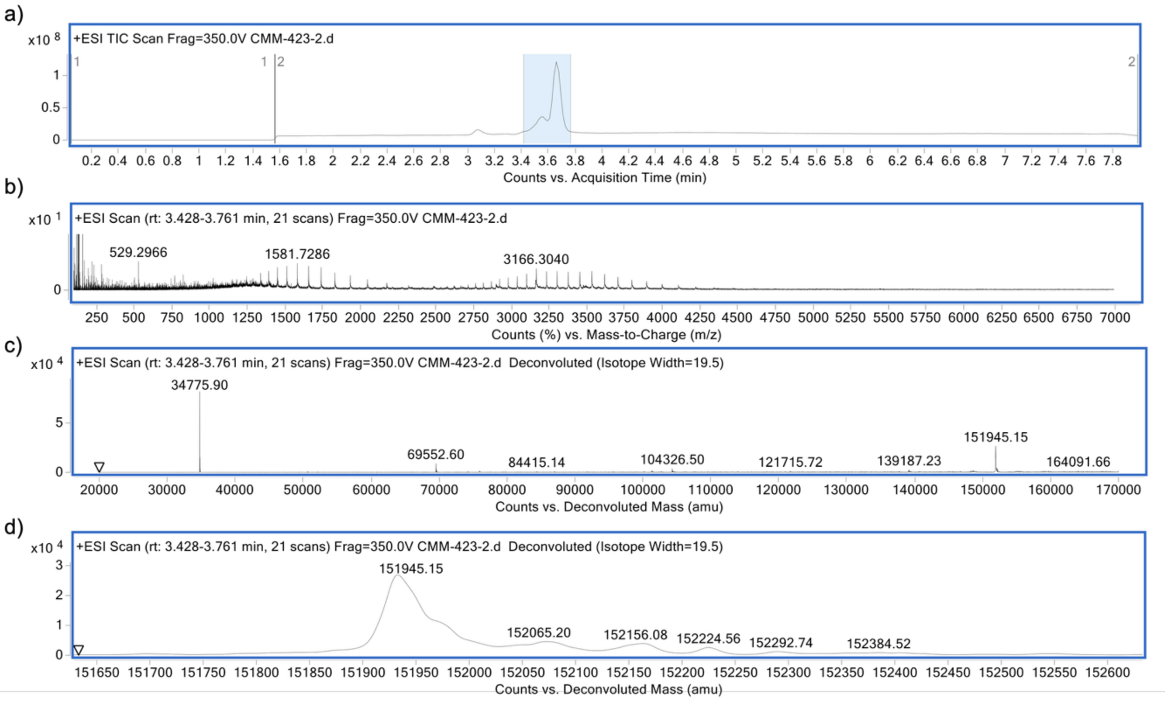

Figure S4. LC-MS spectra for anti-GFP–PD–oligo conjugate **5**, comprising of the (a) total ion chromatogram, (b) non-deconvoluted ion series, (c) deconvoluted ion series mass spectrum, (d) deconvoluted mass spectrum region of interest; observed mass of 151,945 Da corresponds to anti-GFP antibody re-bridged with Tz tetra-PD and clicked with oligonucleotide 5′-TCO-PEG_4-ACCACCACCACCACCA-3′ (calculated mass = 151,983 Da).

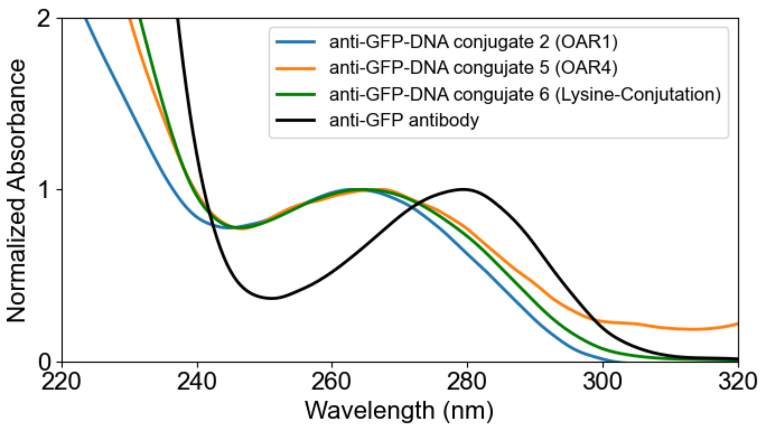

Figure S5. UV-Vis spectra of native anti-GFP antibody (black line), anti-GFP-DNA conjugate **2** (OAR4, orange line), anti-GFP-DNA conjugate **5** (OAR1, blue line), and anti-GFP-DNA conjugate **6** (lysine-directed NHS ester modification, green).

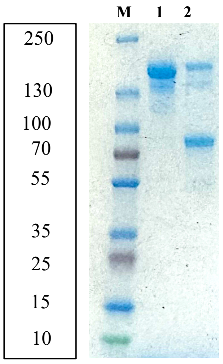

Figure S6. SDS-PAGE gel: M. Ladder, masses listed in kDA, 1. OAR1 conjugate **5**, 2. OAR4 conjugate **2**.

Anti-GFP–lysine–oligo conjugate 6

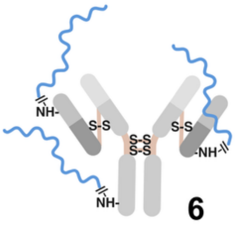

6

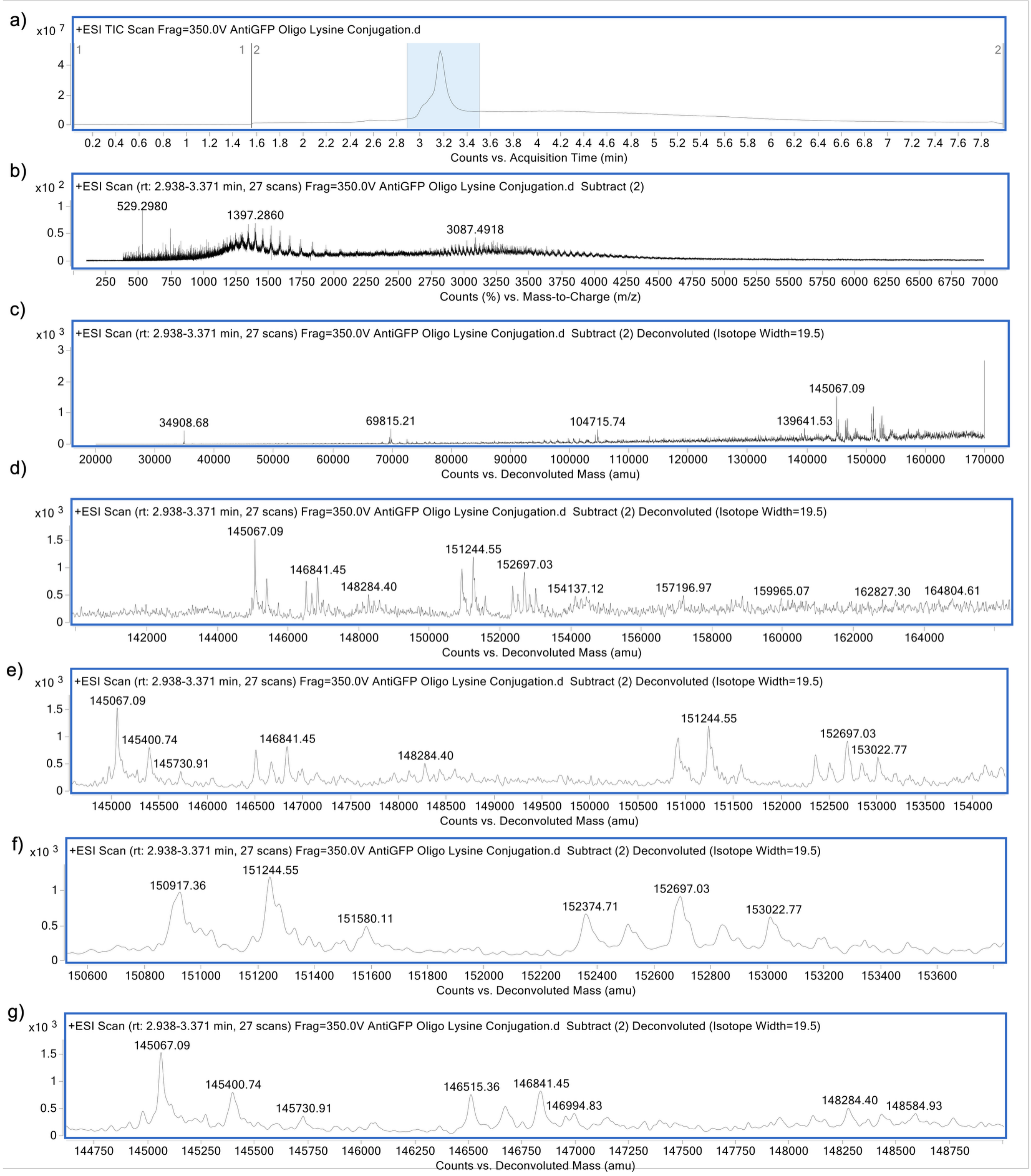

Figure S7. LC–MS spectra for anti-GFP–lysine–oligo conjugate **6** comprising the (a) total ion chromatogram, (b) non-deconvoluted ion series, (c) deconvoluted ion series mass spectrum, and (d–g) deconvoluted mass spectrum regions of interest. A peak corresponding to the native

antibody is observed (observed mass = 145,067 Da; calculated mass = 145,080 Da). A distribution of higher-mass species approximately 4–15 kDa above the native antibody is also observed, consistent with a heterogeneous population of antibody–oligonucleotide conjugates bearing multiple oligonucleotide–linker constructs. Each additional linker contributes ~4.5 kDa, consistent with stochastic lysine-directed NHS ester modification. Peaks at ~35 kDa and higher multiples correspond to PNGase F used during deglycosylation prior to LC–MS analysis. Note that due to the heterogeneous modification strategy generating a plethora of conjugates, the individual ion series are inherently less well defined, and it is unlikely that we would observe all higher loaded antibody–oligonucleotide conjugates by mass spectrometry. UV–Vis analysis gave an average oligonucleotide-to-antibody ratio (OAR) of 3.74.

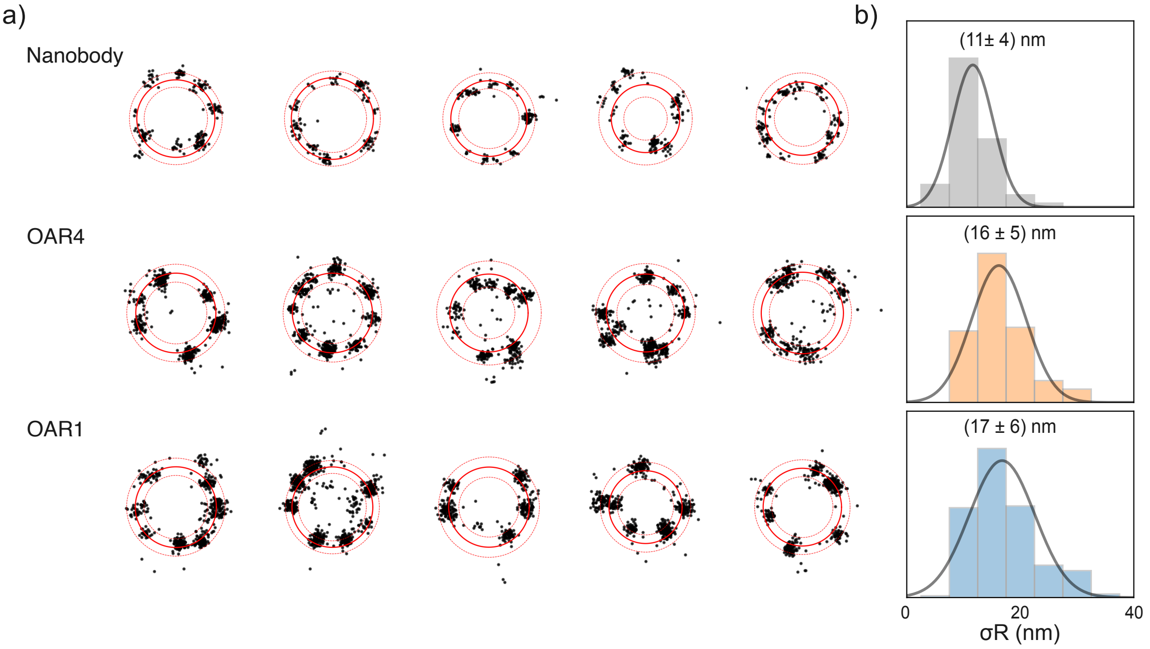

Figure S8. (a) Representative examples of five singles NPCs for each labeling condition, illustrating the dispersion of localizations around the fitted radius. (b) Histograms of the radial spread (σR) measured for each NPC. Mean ± standard deviation values for each labeling condition were obtained by Gaussian fitting of the σR distributions across all NPCs. Antibody-based labeling exhibited a modestly broader radial distribution (σR ~ 16–17 nm) compared to the nanobody probe (σR ~ 11 nm).

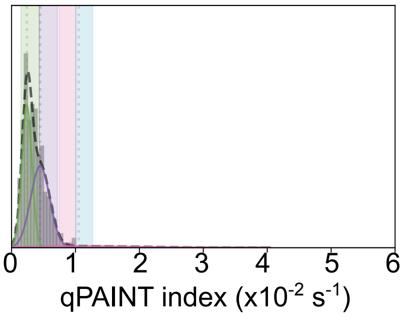

Figure S9. qPAINT index histogram obtained from NPCs in U2OS cells expressing Nup96–GFP, labeled with conventional DNA-functionalized anti-GFP nanobody carrying one speed-optimized DNA strand (F3). qPAINT indices distributions centered at $\mu_1 = 0.24 \times 10^{-2}$ s^{-1} and $\mu_2 = 0.45 \times 10^{-2}$ s^{-1}.

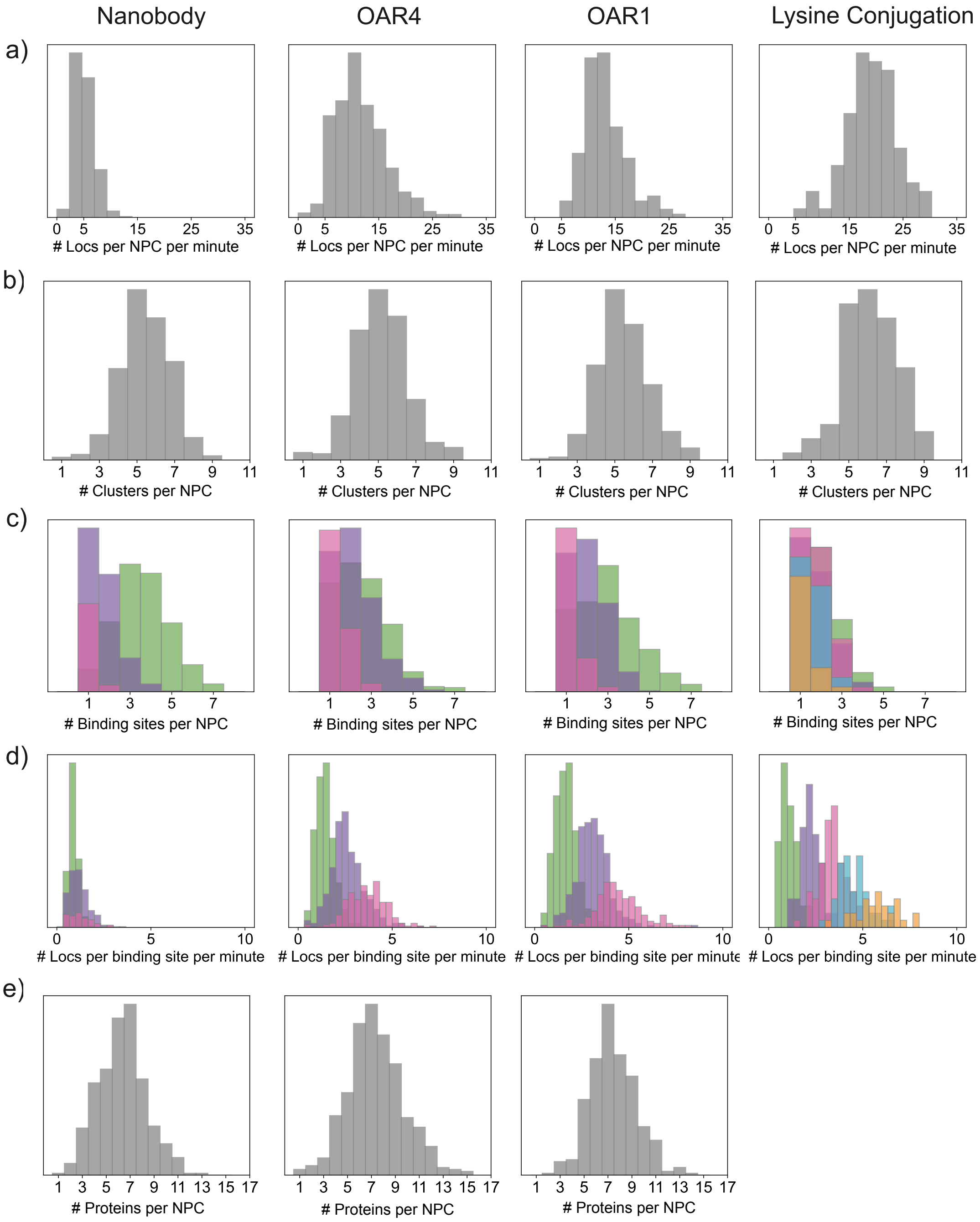

Figure S10. Histograms of measured distributions for each labeling condition (Nanobody, OAR4, OAR1, and random lysine-based labeling). (a) Number of localizations per NPC per minute. (b) Number of clusters per NPC. (c) Number of binding sites per NPC, color-coded by binding-site multiplicity: pink, one binding site; violet, two binding sites; green, three binding sites. Binding-site numbers were derived from qPAINT calibration. (d) Mean number of localizations per binding site per minute. (e) Number of proteins per NPC, calculated from binding-site counts for all conditions except random lysine-based labeling, for which binding sites cannot be uniquely assigned to specific protein numbers.

Supporting References

(1) Bahou, C.; Richards, D. A.; Maruani, A.; Love, E. A.; Javaid, F.; Caddick, S.; Baker, J. R.; Chudasama, V. Highly Homogeneous Antibody Modification through Optimisation of the Synthesis and Conjugation of Functionalised Dibromopyridazinediones. *Org. Biomol. Chem.* **2018**, *16* (8), 1359–1366. https://doi.org/10.1039/C7OB03138F.

(2) Dannheim, F. M.; Walsh, S. J.; Orozco, C. T.; Hansen, A. H.; Bargh, J. D.; Jackson, S. E.; Bond, N. J.; Parker, J. S.; Carroll, J. S.; Spring, D. R. All-in-One Disulfide Bridging Enables the Generation of Antibody Conjugates with Modular Cargo Loading. *Chem. Sci.* **2022**, *13* (30), 8781–8790. https://doi.org/10.1039/D2SC02198F.

(3) Schnitzbauer, J.; Strauss, M. T.; Schlichthaerle, T.; Schueder, F.; Jungmann, R. Super-Resolution Microscopy with DNA-PAINT. *Nat. Protoc.* **2017**, *12* (6), 1198–1228.

(4) Maruani, A.; Savoie, H.; Bryden, F.; Caddick, S.; Boyle, R.; Chudasama, V. Site-Selective Multi-Porphyrin Attachment Enables the Formation of a next-Generation Antibody-Based Photodynamic Therapeutic. *Chem. Commun.* **2015**, *51* (83), 15304–15307. https://doi.org/10.1039/C5CC06985H.

(5) Bryden, F.; Maruani, A.; Rodrigues, J. M. M.; Cheng, M. H. Y.; Savoie, H.; Beeby, A.; Chudasama, V.; Boyle, R. W. Assembly of High-Potency Photosensitizer–Antibody Conjugates through Application of Dendron Multiplier Technology. *Bioconjug. Chem.* **2018**, *29* (1), 176–181. https://doi.org/10.1021/acs.bioconjchem.7b00678.

(6) Endesfelder, U.; Malkusch, S.; Fricke, F.; Heilemann, M. A Simple Method to Estimate the Average Localization Precision of a Single-Molecule Localization Microscopy Experiment. *Histochem. Cell Biol.* **2014**, *141*, 629–638.
